# Epigenetic regulation in early embryogenesis: mechanisms, developmental programming, and transgenerational implications

**DOI:** 10.3389/fcell.2026.1791177

**Published:** 2026-05-08

**Authors:** Igbayilola Yusuff Dimeji, Musafau Oloyede Sanni, Kasim Sakran Abass, Aina Olawale Samson, Abdel Halim Harrath

**Affiliations:** 1 Department of Physiology, College of Medicine, Federal University of Health Sciences, Ila-Orangun, Osun, Nigeria; 2 Department of Nursing Science, Faculty of Allied Health Sciences, Cosmopolitan University, Abuja, Nigeria; 3 Fetal Disease Programming Research Laboratory, Physiology/Molecular and Developmental Biology Unit, Zoology Department, King Saud University, Riyadh, Saudi Arabia; 4 Department of Physiology, Biochemistry, and Pharmacology, College of Veterinary Medicine, University of Kirkuk, Kirkuk, Iraq; 5 Department of Physiology, College of Medicine, Lagos State University, Lagos, Nigeria; 6 Zoology Department, College of Science, King Saud University, Riyadh, Saudi Arabia

**Keywords:** chromatin remodelling, developmental programming, DNA methylation, fetal origins of disease, histone modification, noncoding RNAs

## Abstract

Epigenetics are heritable features of gene expression without changes in the underlying DNA sequence and involve chemical modifications to DNA and histone proteins. The role of these mechanisms is central to the regulation of genes, determination of cell fate, embryonic development, and susceptibility or resistance to disease. Recent advances in low-input and single-cell sequencing technologies have revolutionized understanding of the early embryonic epigenome. These approaches reveal a highly dynamic epigenetic landscape characterized by extensive reprogramming events and stage-specific regulatory factors that preserve transcriptional fidelity while maintaining chromatin integrity. Experimental studies, including human data framed by the Developmental Origins of Health and Disease hypothesis, identify early embryonic and fetal development as a critical window of biological sensitivity. Adverse environmental conditions, such as maternal undernutrition, metabolic dysfunction, or other physiological stressors, may disrupt epigenetic reprogramming during this window. These could affect fetal growth, as well as provoke perpetual changes in the regulation of hormones, growth, as well as response of tissues. Thus, those persons who were exposed to less-than-optimal conditions during fetal growth face a higher risk of having perpetual disorders during their lifetime, including type 2 diabetic mellitus, cardiovascular disease, as well as disorders of metabolic syndromes. Although great progress has been made, there are still several important questions related to epigenetic marks that have been shown to avoid erasure during embryonal reprogramming. This review gathers available data regarding the control of early developments by epigenetics.

## 1 Introduction

Epigenetic regulation has become the cornerstone of modern developmental biology, giving insight into the functional interpretation of the genome without changes in DNA sequence (Kakoulidou et al., 2021). The concept of an epigenetic landscape defines DNA methylation, histone modifications, chromatin remodeling, and higher-order chromatin arrangement; all these processes together regulate gene expression with a high-degree of spatial and temporal precision (Cui et al., 2025). Such processes begin to operate in the earliest model stages in the developmental lifespan cycle among embryos and represent a fundamental force in cell fate determination decisions and even intergenerational transmission in certain models (Cheedipudi et al., 2014).

Of the fundamental epigenetic processes, DNA methylation, which is largely targeted to CpG dinucleotides, is crucial to early mammalian development. Indeed, the DNA methylation landscape of the mammalian embryo is highly dynamic and extensive, involving massive erasure and reimprinting cycles (Adetunji et al., 2025; Huang et al., 2019). A massive genome-wide erasure of methylation marks takes place in the primordial germ cells. In these cells, the erasure of methylation marks is crucial to the prevention of the germline transmission of somatic epimutations (Tompkins, 2023). This is achieved through both passive and active processes that involve DNA replication, dilution mechanisms mediated by ten-eleven translocation (TET) enzymes, as well as the participation of DNA replication-based base-excision repair mechanisms (Zeng and Chen, 2019). After erasure, the DNA methylation imprint is reimprinted in the male and female germline using the *de novo* DNA methyltransferases DNMT3A and DNMT3B aided by the accessory factor DNMT3L to create the male and female DNA methylation imprinting profiles (Gujar et al., 2019). The second major wave of epigenetic rewriting happens shortly after fertilization. Here, the paternal genome is subject to a process of active demethylation, while the maternal genome is subject to progressive demethylation in a passive manner in the course of subsequent cell divisions (Singh et al., 2023). Importantly, this global rewriting process excludes imprinted genes and defined subgroups of retrotransposons, thus maintaining genes’ parent-of-origin-dependent methylation patterns, which are critical in the course of embryo development (Fang et al., 2024). As the implantation phase is about to be initiated, the process of *de novo* methylation is started once more to set the epigenetic lineage patterns critical in the process of initial tissue differentiation and organogenesis (Guo and Yang, 2024).

Aside from DNA methylation, histone-mediated processes are major factors in epigenetic development regulation. Along with histone H3 and H4 post-translational modifications, histone variant incorporation has been shown to differently regulate nucleosome structure and stability, as well as interactions with chromatin remodeling proteins (Huang Z. et al., 2025). These processes are then processed by cellular reader proteins that decode chromatin structure and dynamics coupled with TFs, cellular signals, and long-range chromatin structures for refining developmental gene expression regulation (Oksuz et al., 2023; Yang et al., 2025). Chromatin remodeling proteins such as SWI/SNF chromatin remodeling ATPase engines, CHD chromatin remodeling proteins, ISWI chromatin remodeling proteins, and INO80 chromatin remodeling proteins introduce a further layer of complexity with the use of ATP for repositioning, removing, or reorganizing nucleosomes from gene structures for development regulation purposes (Huang Y. et al., 2025; Prajapati et al., 2020). The roles of chromatin remodeling proteins are indispensable for processes such as zygote genome activation cycles, pluripotency phases, DNA damage repair processes, and developmental lineage specification events (Dabin et al., 2016).

Importantly, epigenetic regulation is not strictly an embryonic process because the impact of epigenetic events could extend to the next-generation. While a high level of epigenetic reprogramming removes most epigenetic marks that were transmitted from the parental lineage, some could remain intact or revert, making it feasible for the occurrence of transgenerational epigenetic inheritance (Greenberg and Bourc’his, 2019; Wilkinson et al., 2023). This could result from the interaction of environmental stress and/or metabolic disruptions affecting germline epigenetic patterns, resulting in lasting effects in the next generations with regard to either stress responsiveness or metabolism and/or diseases. Inheritance could result from the formation and maintenance of DNA methylation patterns, histone patterns, and/or small RNA-mediated systems whose impact is to overcome and/or reinitiate epigenetic programming (Quarato et al., 2022). Despite major advances in understanding epigenetic regulation during early embryogenesis, the field is still characterized by rapidly expanding, sometimes fragmented evidence, spanning DNA methylation dynamics, histone modifications, chromatin remodeling, and higher-order genome organization. The extensive epigenetic reprogramming events occurring in germ cells and preimplantation embryos are integral to normal development, lineage specification, and imprinting (Lan and Shi, 2009; Du et al., 2022). Precisely how these processes are coordinated and their vulnerability to environmental perturbations remain incompletely resolved. Furthermore, emergent evidence that certain epigenetic marks can evade reprogramming or become re-established emphasizes the potential for transgenerational inheritance of disease risk and phenotypic traits, with important biological and public health implications (Burggren, 2016; Fallet et al., 2023). This review is therefore timely and necessary to integrate the current knowledge on epigenome maintenance and reprogramming during early development, clarify mechanistic interplay between DNA methylation, histone dynamics, and chromatin remodeling, and critically evaluate the emerging evidence linking early epigenetic regulation to long-term developmental outcomes and intergenerational effects.

## 2 Methods

### 2.1 Method of literature search

A structured literature search was conducted to retrieve peer-reviewed literature relevant to epigenetic regulation in early embryogenesis, developmental programming, and transgenerational inheritance. Literature search engines such as PubMed/MEDLINE, Web of Science, Scopus, and Google Scholar were used. Primarily, literature from January 2015 to December 2025 was searched, as this period has seen tremendous advances in epigenomic technologies and mechanistic understanding.

Literature search was conducted on 15 December 2025. Literature search terms included “epigenetic regulation,” “early embryogenesis,” “DNA methylation,” “histone modification,” “chromatin remodeling,” “non-coding RNA,” “developmental programming,” “fetal origins of disease,” “transgenerational epigenetic inheritance,” etc. These terms were searched separately and in combination using Boolean logic operators AND/OR.

Literature search results included peer-reviewed original research articles and review articles from experts on epigenetic regulation in early embryonic development in humans and mammalian species. Literature search results were selected based on their ability to provide insights into epigenetic regulation in early embryos and their ability to provide mechanistic insights into epigenetic regulation in early embryos. Literature search results that lack sufficient methodology, experimental design, and relevance to epigenetic regulation in embryos were excluded.

While the major emphasis was placed on publications after 2015, other landmark publications published before 2015 were included when necessary to provide historical backgrounds or to provide foundational mechanistic knowledge. This was done through citation tracking of major publications and review articles.

**FIGURE 1:**
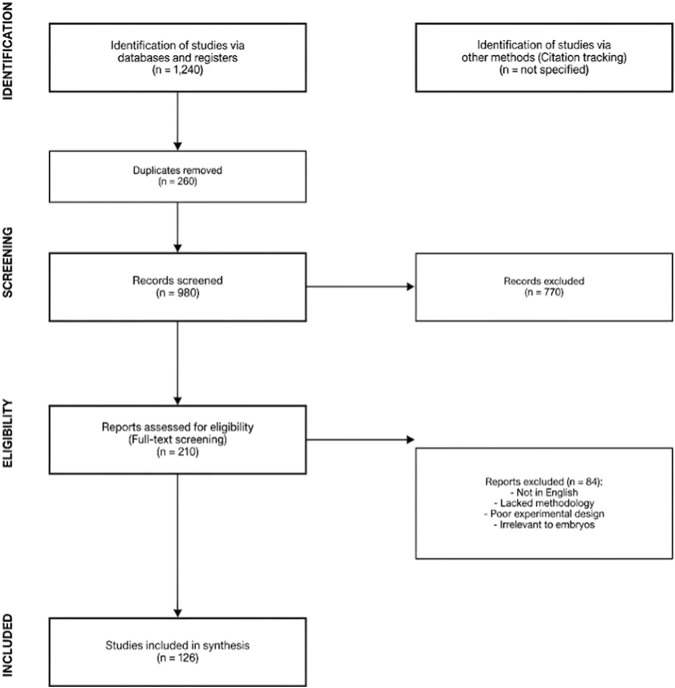
Flow chart in the PRISMA style that summarises the process of finding, screening, determining a study’s eligibility, and ultimately adding it to the review.

The search of the database resulted in approximately 1,240 publications. Following the elimination of duplicates, 980 publications were identified as potential sources of information. Out of these, 210 publications were selected through full-text screening based on relevance and quality of the publications, resulting in 126 publications being included in the final synthesis. The publications selected were those written in English language only. The selection of publications was done following the guidelines of PRISMA, and a simple diagram of the selection process is shown in the manuscript. See Figure 1.

## 3 Epigenetic reprogramming in early embryos

### 3.1 DNA methylation

**FIGURE 2:**
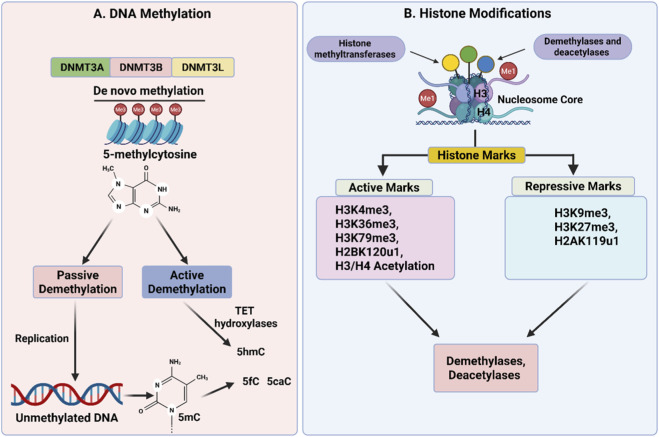
Epigenetic reprogramming in early embryos. **(A)** DNA methylation: Using DNMT3L as a cofactor, DNMT3A and DNMT3B create *de novo* 5-methylcytosine (5mC) at CpG sites. Active demethylation is mediated by TET enzymes via 5hmC, 5fC, and 5caC intermediates. **(B)** Histone modifications: Marks linked to repressive (H3K9me3, H3K27me3, H2AK119u1) or active (H3K4me3, H3K36me3, H3K79me3, H2BK120u1, H3/H4 acetylation) chromatin are added by histone methyltransferases and acetyltransferases. These marks are dynamically altered by demethylases and deacetylases.

DNA methylation is an epigenetic modification involving the addition of a methyl group to the 5-position of cytosine, giving rise to 5-methylcytosine (5mC). DNA methylation occurs mainly in CpG sites. *De novo* DNA methylation is carried out by DNMT3A and DNMT3B, with DNMT3L acting as a non-catalytic cofactor. DNA methyltransferase 1 maintains DNA methylation. In the absence of DNA methyltransferases, 5mC can be lost due to passive dilution during DNA replication or can be actively removed by TET family enzymes, which convert 5mC to 5-hydroxymethylcytosine (5hmC) and further to 5-formylcytosine and 5-carboxylcytosine (Figure 2).

DNA methylation is dynamically reprogrammed in early embryonic development. Global demethylation is carried out in waves to return parental epigenetic marks to their original status, leading to totipotency (Arand et al., 2021; Fulka et al., 2004; Petrussa et al., 2016). The first major wave of global demethylation occurs within 12 h after fertilization, focusing on intergenic and gene body methylation (Guo et al., 2014; Smith et al., 2014); additional global demethylation takes place from zygote to 2-cell stages, as well as from 8-cell stages to blastocysts, focusing on introns and young SINEs (Zhou et al., 2019; Zhu et al., 2018). TET3 is highly expressed in oocytes, consistent with 5hmC accumulation at newly demethylated sites (Gu et al., 2011; Yan et al., 2023).

In terms of methylation, there are differences in the paternal and maternal genomes. Sperm is very methylated, but in oocytes, methylation is lower. However, after fertilization, there is rapid demethylation in the paternal genome, but maternal methylation is sustained for a longer period, resulting in parental origin effects and differentially methylated regions (Guo et al., 2014; Smith et al., 2014; Arand et al., 2021; Fulka et al., 2004). Some maternal methylation persists until blastocyst and placental stages (Hanna et al., 2016; Sanchez-Delgado et al., 2016).

Imprinted genes maintain allele-specific expression by gametically established methylation, which is protected in primary imprinting control regions by factors such as ZNF445 (Ishida and Moore, 2013). Other protected regions include young LINE-1 elements, which prevent their activation (Takahashi et al., 2019). *De novo* methylation is a transient phenomenon from 4 to 8 cell stages, targeting SINE, LINE, and LTR sequences, but many are also removed in morula and blastocyst stages (Ivanova et al., 2020; Wilkinson et al., 2023). This is important for totipotency, cell type specification, and normal embryo development. Any alterations in this process will lead to developmental problems, imprinting disorders, or lethality (Greenberg and Bourc’his, 2019; Li et al., 2017).

### 3.2 Histone modifications

Histones are responsible for forming the nucleosomes, which are important in DNA organization. Histones are also important in controlling chromatin accessibility. A nucleosome is composed of a tetramer formed by histones 3 and 4, along with dimers formed by histones 2A and 2B. These histones are wrapped by 147 base pairs of DNA (Suganuma and Workman, 2011). Histones have tails that are important in various modifications such as acetylation, methylation, phosphorylation, ubiquitylation, and sumoylation (Zhang et al., 2015; Suganuma and Workman, 2011).

**FIGURE 3:**
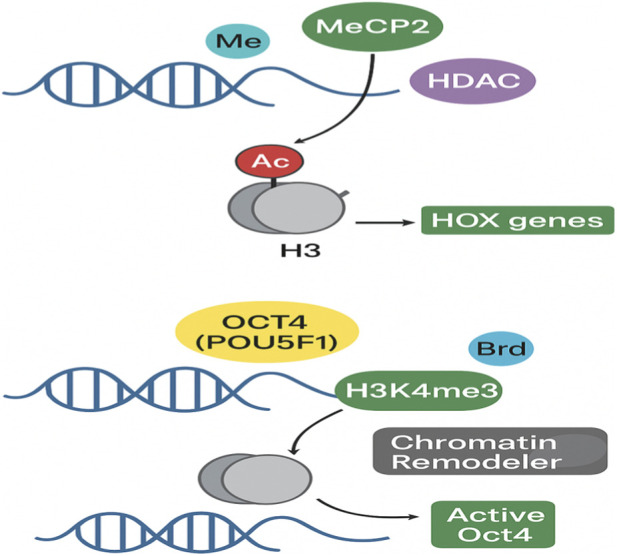
Epigenetic crosstalk in gene expression regulation during embryogenesis.

Histone modifications such as H3K4me3, H3K36me3, H3K79me3, H3/H4 acetylation, and H2BK120u1 are linked to active transcription, whereas H3K9me3, H3K27me3, and H2AK119u1 indicate restrictive chromatin (Zhang et al., 2015; Margueron and Reinberg, 2011). Histone modifications are regulated by writers, such as methyltransferases and acetyltransferases, and erasers, such as demethylases and deacetylases (Hodawadekar and Marmorstein, 2007; Yang and Seto, 2007) (Figure 3). The chromatin structure becomes loose when histones are acetylated because the lysine residue’s positive charge is removed (Zhang et al., 2015; Margueron and Reinberg, 2011).

Histone modifications play a critical role in regulating gene expression during embryonic development. Embryonic stem cell chromatin is marked by a unique histone modification pattern, including bivalency (H3K4me3 and H3K27me3) at key gene promoters. These histone modifications are required for gene expression during differentiation (Azuara et al., 2006; Be, 2006).

Reprogramming in primordial germ cell and embryo development is characterized by decreased H3K9me2 and increased H3K4me2/3 and H3K27me3. These histone modifications are required for totipotency (Gan et al., 2007; Hemberger et al., 2009). Following fertilization, there is a decrease in H3K4me3 on the paternal genome, which is later replenished after zygotic genome activation. During this time, there is a decrease in H3K27me3 on the maternal genome in specific regions. These histone modifications are required for gene expression during early development in concert with DNA.

For example, methylated CpG sites are bound by methyl-CpG-binding proteins, especially MeCP family proteins such as MeCP2, which recruit transcriptional corepressor complexes such as histone deacetylases and histone methyltransferases, resulting in condensation of chromatin and transcriptional repression (Fuks et al., 2003; Nan et al., 1998). However, histone acetylation can resist the action of DNA methylation. HDAC inhibitors, “valproate and trichostatin A” provide insights into this histone alteration action and regulation (Dong et al., 2007; Ou et al., 2007).

The epigenetic control of embryonic genes is depicted in this figure. Histone deacetylases and methyl-CpG-binding proteins are drawn to DNA methylation, which compacts chromatin to suppress HOX genes. OCT4 and other pluripotency genes maintain active histone marks, which draw chromatin remodelers to sustain transcription.

## 4 Transgenerational epigenetic inheritance

Transgenerational epigenetic inheritance (TEI) is defined as “the transmission of phenotypes from one generation to the next through epigenetic means instead of DNA sequence changes.” Epigenetic modifications such as DNA methylation, histone modification, and non-coding RNA play important roles in gene expression and are passed on from one generation to another via the germline (Fitz-James and Cavalli, 2022).

In invertebrates and plants, TEI has been extensively studied, but in mammals, there is limited information. In *Caenorhabditis*, RNA-mediated epigenetic inheritance is responsible for conferring adaptive pathogenic resistance in a Darwinian-like gene expression system, which is responsive to environmental conditions (Ashe et al., 2012). In *Schizosaccharomyces pombe*, transient gene silencing via heterochromatin confers protection against environmental stress, which is relieved once stress is alleviated (Torres-Garcia et al., 2020).

**FIGURE 4:**
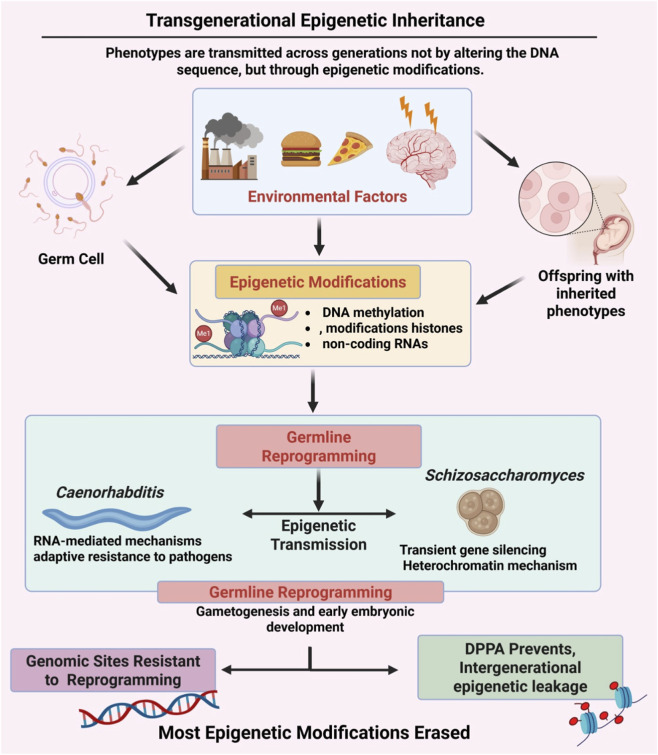
Transgenerational epigenetic inheritance. In transgenerational epigenetic inheritance, the environment, including pollutants, nutrition, and stress, affects DNA methylation, histone modifications, and non-coding RNAs. The majority of epigenetic marks are reset to the default state through gametogenesis, but some marks allow for intergenerational inheritance.

The major hindrance to TEI in mammals is epigenetic reprogramming in the germline, which removes DNA methylation and histone marks in the majority of the genome during gametogenesis and early development (Luo et al., 2018). However, some regions of the genome, like metastable epialleles in mice, have been resistant to epigenetic reprogramming, retaining DNA methylation in the genome (Luo et al., 2018). Protective factors like DPPA2 also act as hindrances to intergenerational epigenetic leakage (Carlini et al., 2022) (Figure 4).

Environmental compounds, including dietary, stress, and lifestyle factors, have been tested for their TEI potential. In rodents, paternal dietary restriction or exposure to endocrine disruptors has been reported to cause transient changes in offspring, affecting metabolism and reproduction (Carone et al., 2010; Schneider et al., 2013). Alteration of the paternal microbiome has also been reported to cause disruptions in placental development and offspring viability, independent of sperm epigenome changes (Argaw-Denboba et al., 2024).

For mammals, the phenotypic effects of transgenerational epigenetic inheritance (TEI) include changes in metabolism, behavior, and disease susceptibility (Rando, 2012). In humans, TEI is supported by epidemiological data, but it is not universally accepted. The impact of historical events such as famine in Sweden and trauma from the Holocaust may have had transgenerational effects, but it is difficult to distinguish these from cultural and behavioral effects. The idea of inherited metabolic traits such as obesity is controversial and may be compared with classical evolutionary adaptations such as malaria resistance in those heterozygous for sickling hemoglobin. The phenomenon of TEI is plausible but not supported by robust data in mammals and humans. Most effects appear transient, weak, and highly dependent on context. Advances in genetics, such as mutation tracking in developmental and adult traits, have offered alternative explanations for environmental epigenetics effects.

### 4.1 Specification and reprogramming of germ cells

Though generally somatic cells have relatively stable epigenetic patterns during adult life, achieving totipotency requires extensive epigenome reprogramming during development; likewise, global reprogramming of germ cells occurs during embryogenesis, and the germline epigenetic signatures are preserved at least until later stages of differentiated cells-such as spermatocytes or spermatids-despite the postnatal epigenetic changes. Epigenetic inheritance needs to reconcile with such reprogramming events, which are necessary for organogenesis and germ cell production. The first significant reprogramming in mammals takes place at fertilization, resulting in the alteration of the zygote’s chromatin architecture; leading to the formation of the germ layers (Bondarieva and Tachibana, 2024); this is followed by the transition from somatic-to-germline at E6.5 to E13.5 and a male-specific additional event during spermatogenesis. Reprogramming of the genome needs BLIMP1/PRDM1 that cooperates with PRMT5 and LSD1 to regulate histone modifications and repress somatic genes, whereas PRDM14 and AP2γ activate pluripotency (Hackett and Surani, 2014; Magnúsdóttir et al., 2013). Human ESC-derived hPGCLCs use SOX17 and BLIMP1 to confer the germ cell fate and cytokines FGF2 and TGF-β to impose differential pluripotency compared to mouse (Eguizábal et al., 2016; Gkountela et al., 2013; Irie et al., 2015). Genes controlling gametogenesis, such as DAZL, SYCP1-3, and RAD51C, are epigenetically regulated to ensure appropriate germline function. The accurate epigenetic resetting during spermatogenesis is also essential for achieving totipotency and for avoiding transgenerational defects.

### 4.2 Environmentally induced epigenetic mutations

#### 4.2.1 Environmental influences

While the structure of DNA itself does not change, environmental factors, including diet, can affect DNA methylation patterns across generations. *In utero* exposure to a high-fat diet can cause paternal obesity and insulin resistance, coupled with sperm microRNA content and changes in the methylation of germ cells from two successive generations (Fullston et al., 2013). Similarly, intrauterine hyperglycemia leads to metabolic intergenerational changes in the F2 but not the F3 generation (Ren et al., 2018). Most notably, chemical exposures, specifically the Endocrine disrupting chemicals (EDCs), represent significant threats to epigenetic integrity. EDCs are found in pesticides, food packaging, and personal care products and are able to perturb endocrine signaling and disrupt long-term health. Studies have shown this to be true in (Anway et al., 2005; WHO, 2017). Transgenerational effects, however, can only be determined if rodent studies examine the F3 generation-the first to be unaffected by direct toxicant exposure. F0 pregnant females are exposed during all embryonic reprogramming stages (E6–E15), and the F1 fetuses are also directly exposed. The F2 and F3 generations are generated via breeding of non-littermate animals to assure F3 does indeed represents unexposed descendants. Work in the Skinner laboratory showed that exposure to a variety of chemicals during this critical window produced transgenerational effects in F3 and F4 generations characterized by changes in sperm DNA methylation (Manikkam et al., 2014; Manikkam et al., 2012; Manikkam et al., 2013; Skinner et al., 2013; Tracey et al., 2013). Pregnancy exposure to tributyltin predisposed F3 offspring to obesity through chromatin organization and methylation changes (Diaz-Castillo et al., 2019). Similarly, atrazine and chlordecone induced histone H3K4 trimethylation changes and transgenerational epigenetic modifications (Gely-Pernot et al., 2018; Hao et al., 2016). These studies suggest that environmental exposures can exert long-lasting effects on epigenetic regulation with consequences for health over multiple generations. Table 1 presents several recent studies demonstrating how environmental factors induce transgenerational effects on epigenetic marks in rodent species.

**TABLE 1:** Examples of intergenerational and transgenerational epigenetic associations in mammals.

Epigenetic mark affected	Environmental factor	Organ/Matrix	Animal model	First unexposed generation tested	Type of effect	Associated health outcome	References
Hypomethylation of Il13ra2 and altered expression of 642 pancreatic islet genes	Paternal high-fat diet	Pancreatic islets	Sprague–Dawley rats	F2	Transgenerational	Impaired glucose–insulin homeostasis (Type 2 diabetes)	Ng et al. (2010)
∼20% change in cytosine methylation at PPARα enhancer	Paternal low-protein diet	Liver	C57BL/6 mice	F2	Transgenerational	Altered cholesterol and lipid metabolism	Carone et al. (2010)
Altered insulin-2 promoter and reduced PDX1 binding	Multigenerational undernutrition	Pancreas	Wistar rats	F2	Intergenerational	Adiposity/Type 2 diabetes	Hardikar et al. (2015)
↓ H3K9 acetylation and ↑ H3K9 dimethylation at adiponectin and leptin promoters	Maternal high-fat diet	Adipose tissue	ICR mice	F2	Intergenerational	Obesity and impaired glucose homeostasis	Masuyama et al. (2016)
Differentially methylated genes detected in F2 but not F3	Intrauterine hyperglycemia	Primordial germ cells	ICR mice	F2	Intergenerational	Obesity, insulin resistance	Ren et al. (2018)
Changes in DNMT1, Mecp2, Hdac1 and histone acetylation	Diet restriction	Liver, adipose, muscle	Wistar rats	F2	Intergenerational	Metabolic alterations	Nowacka-Woszuk et al. (2018)
Hypo- and hypermethylation of MeCP2, CB1, CRFR2	Chronic maternal separation	Brain, sperm	C57BL/6J mice	F2	Transgenerational	Depressive-like behavior	Franklin et al. (2010)
↑ DNMT1, TET1, ↓ 5mC/5hmC at Bdnf	Prenatal restraint stress	Brain	Swiss albino mice	F2	Intergenerational	Schizophrenia-like phenotype	Dong et al. (2007)
miRNA dysregulation (miR-103, miR-145, miR-323, miR-98, miR-219)	Maternal stress (swim/restraint)	Brain	Long-Evans rats	F2	Intergenerational	Neuropsychiatric-like phenotypes	Zucchi et al. (2013)
CpG hypomethylation at Olfr151	Olfactory fear conditioning	Sperm	C57BL/6J, M71-LacZ mice	F1	Intergenerational	Fear and behavioral sensitivity	Dias and Ressler (2014)
↓ H4K5 and H3K14 acetylation; altered MR gene histone marks; ↑ sperm DNA methylation and miR-375	Maternal separation/stress	Hippocampus, sperm	C57BL/6 mice	F2	Transgenerational	Anxiety-like behavior	Gapp et al. (2014)
↓ Methylation of AT1b receptor gene	Maternal low-protein diet	Adrenal gland	Wistar rats	F2	Intergenerational	Hypertension	Bogdarina et al. (2007)
Altered CpG methylation in dendritic cells	Maternal intranasal particles	Dendritic cells	BALB/c mice	F3	Transgenerational	Asthma susceptibility	Gregory et al. (2017)
Gene expression changes in hippocampus and amygdala	Maternal vinclozolin exposure	Brain	Sprague–Dawley rats	F3	Transgenerational	Anxiety-like behavior	Skinner et al. (2008)
Altered methylation of H19, Gtl2, Peg1, Snrpn, Peg3	Vinclozolin injection	Sperm	FVB/N mice	F2	Transgenerational	Reduced sperm concentration	Stouder and Paoloni-Giacobino (2010)
miRNA and HIF-1α alterations in lung	Second-hand cigarette smoke	Lung	BALB/c mice	F2	Intergenerational	Asthma and bronchopulmonary dysplasia	Singh et al. (2017)
Increased DNA methylation in sperm across F1–F3	Maternal atrazine exposure	Sperm	Sprague–Dawley rats	F3	Transgenerational	Lean phenotype, hyperactivity	McBirney et al. (2017)
Differentially methylated sperm promoters (197 regions)	Maternal exposure to plastic compound mixture (BPA, DEHP, DBP)	Sperm	Sprague–Dawley rats	F3	Transgenerational	Obesity and sperm abnormalities	Manikkam et al. (2013)

In this table, intergenerational effects mean the effects seen in the directly exposed offspring (F1) or in the F2 generation, provided that the developing germline in the fetus was impacted by the maternal exposure. Transgenerational effects mean the phenotypes seen in the generations that were not directly exposed to the environmental factor, usually in the F3 generation after maternal exposure or in the F2 generation after paternal exposure.

### 4.3 Examples of phenotypic consequences in offspring

#### 4.3.1 Metabolic phenotypic effects

Parental and fetal environmental exposures can have profound effects on metabolic phenotypes. Evidence from animal and human research indicates that several factors, including maternal obesity, high-fat and low-protein diet, endocrine disruptors, and prenatal stress, result in offspring with an elevated risk of obesity, insulin resistance, and glucose intolerance (Dietz et al., 2011; Feeney et al., 2014; Holuka et al., 2024; Huypens et al., 2016; Igbayilola et al., 2021; Masuyama et al., 2016; Rodgers et al., 2013; Soubry, 2015). These effects are often seen to be enduring throughout life and even transmitted intergenerationally. Various abnormalities of lipid metabolism, such as hypertriglyceridemia and hypercholesterolemia, as well as energy metabolism and mitochondrial function, have been widely observed. Macronutrients, like fats and proteins, and micronutrients, in particular vitamins, are suggested by many *in vitro* studies to affect epigenetic regulation. Nutrients from the diet can affect immune and developmental processes via four major epigenetic mechanisms (Figure 2). First, certain nutrients act as methyl or acetyl donors or as cofactors for enzymes involved in DNA methylation and histone modifications, modulating genes related to growth and immunity (Figures 2, 3). For example, folic acid, choline, betaine, and methionine contribute to one-carbon metabolism, which directly supports DNA methylation and histone modification, affecting gene expression in relation to metabolism of lipids and glucose and immune function and nucleic acid metabolism. Folic acid, methionine, and other one-carbon metabolism–related metabolites restricted during pregnancy reduced CpG island methylation of the offspring genome, as reported by Sinclair et al. (2007), potentially causing preterm birth and abnormal development. Timely folic acid supplementation was able to reverse these effects through modifications to genomic DNA methylation. It highlights an ever-present risk for developing metabolic disorders like metabolic syndromes and type 2 diabetes and an accompanying risk for developing obesity. See Tables 2, 3 for current epigenetic inheritance research and data using in experimental animal and human models respectively.

**TABLE 2:** Environmental epigenetics and epigenetic inheritance in domestic farm animals. From: epigenetics and transgenerational inheritance in domesticated farm animals.

Species	Finding	Context	References
Bovine	Mammary gland-specific hypomethylation of Hpa II sites flanking the bovine alpha S1-casein gene	Epigenetic regulation of lactation	Platenburg et al. (1996)
	DNA-remethylation around a STAT5-binding enhancer in the alphaS1-casein promoter is associated with abrupt shutdown of alphaS1-casein synthesis during acute mastitis	Epigenetic regulation of lactation	Vanselow et al. (2006)
	Transcriptome profiling of *Streptococcus* uberis-induced mastitis reveals fundamental differences between immune gene expression in the mammary gland and in a primary cell culture model	Epigenetic regulation of lactation	Swanson et al. (2009)
	Conservation of methylation reprogramming in mammalian development: aberrant reprogramming in cloned embryos	Epigenetic changes with assisted reproductive technologies	Dean et al. (2001)
	DNA methylation events associated with the suppression of milk protein gene expression during involution of the bovine mammary gland	Epigenetic regulation of lactation	Singh et al. (2023)
	Effect of maternal lactation during pregnancy	Epigenetic regulation of lactation	Gonzalez-Recio et al. (2012)
	Large offspring syndrome in cattle and sheep	Epigenetic changes with assisted reproductive technologies	Young et al. (1998)
	The production of unusually large offspring following embryo manipulation: Concepts and challenges	Epigenetic changes with assisted reproductive technologies	Walker et al. (1996)
	Postnatal characteristics of calves produced by nuclear transfer cloning	Epigenetic changes with assisted reproductive technologies	Garry et al. (1996)
	Epigenetic contribution to individual variation in response to lipopolysaccharide in bovine dermal fibroblasts	Epigenetic role in immunity	Green and Kerr (2014)
	Occurrence, absorption and metabolism of short chain fatty acids in the digestive tract of mammals	Epigenetic changes with regulation of nutrition	Bugaut (1987)
	Butyrate induces profound changes in gene expression related to multiple signal pathways in bovine kidney epithelial cells	Epigenetic changes with regulation of nutrition	Li and Li (2006)
	Transcriptome Characterization by RNA-seq Unravels the Mechanisms of Butyrate-Induced Epigenomic Regulation in Bovine Cells	Epigenetic changes with regulation of nutrition	Wu et al. (2012)
	*In vitro* produced and cloned embryos: Effects on pregnancy, parturition and offspring	Epigenetic changes with assisted reproductive technologies	Kruip and Den Daas (1997)
Porcine	Sulforaphane causes a major epigenetic repression of myostatin in porcine satellite cells	Histone deacetylase inhibitor affects myostatin *in vitro*	Fan et al. (2012)
	Maternal dietary protein affects transcriptional regulation of myostatin gene distinctively at weaning and finishing stages in skeletal muscle of Meishan pigs	Maternal diet affects offspring epigenetics	Liu et al. (2011)
	HOX10 mRNA expression and promoter DNA methylation in female pig offspring after *in utero* estradiol-17beta exposure	Maternal steroid exposure affects offspring epigenetics	Pistek et al. (2013)
	Investigations on transgenerational epigenetic response down the male line in F2 pigs	Paternal diet has transgenerational epigenetic effect	(Braunschweig et al., 2012)
	Dietary Sulforaphane, a Histone Deacetylase Inhibitor for Cancer Prevention	Epigenetic changes with regulation of nutrition	Ho and Crabtree (2010)
	Neonatal estradiol exposure alters uterine morphology and endometrial transcriptional activity in prepubertal gilts	Steroid exposure affects epigenetics	Tarleton et al. (2001)
	Maternal dietary protein restriction and excess affects offspring gene expression and methylation of non-SMC subunits of condensin I in liver and skeletal muscle	Maternal diet affects offspring epigenetics	Altmann et al. (2012)
	Diet, methyl donors and DNA methylation: interactions between dietary folate, methionine and choline	Epigenetic changes with regulation of nutrition	MD (2002)
Ovine	Periconceptional nutrition and the early programming of a life of obesity or adversity	Maternal diet affects offspring epigenetics	Zhang et al. (2011)
	The effect of maternal under-nutrition before muscle differentiation on the muscle fiber development of the newborn lamb	Maternal diet affects offspring epigenetics	Fahey et al. (2005)
	Effect of maternal dietary restriction during pregnancy on lamb carcass characteristics and muscle fiber composition	Maternal diet affects offspring epigenetics	Daniel et al. (2007)
Gallus	Comparison of the Genome-Wide DNA Methylation Profiles between Fast-Growing and Slow-Growing Broilers	Differences in epigenetics between breeds	Hu et al. (2013)
	Insulin-like growth factor-1 receptor is regulated by microRNA-133 during skeletal myogenesis	Epigenetic effects during muscle development	Huang et al. (2011)
	Transgenerational epigenetic effects on innate immunity in broilers: An underestimated field to be explored?	Review on role of epigenetics in innate immunity	Berghof et al. (2013)
	DNMT gene expression and methylome in Marek’s disease resistant and susceptible chickens prior to and following infection by MDV.	Epigenetic role in immunity	Tian et al. (2013)
Porcine	Investigations on transgenerational epigenetic response down the male line in F2 pigs	Paternal diet has transgenerational epigenetic effect	Braunschweig et al. (2012)

**TABLE 3:** Overview of selected studies on intergenerational (up to F1) and transgenerational (F2 and beyond) inheritance.

Category	Stressor/Exposure	Species	Exposed sex (F0)	Offspring phenotype	Proposed biological mechanism	Persistence (generations)	References
Physiological (Nutrition)	Hunger/Nutritional deprivation (during pregnancy)	Human	Female	Cardiovascular diseases; impaired glucose tolerance; obesity and type 2 diabetes	DNA methylation (decrease in IGF2 gene methylation)	F2	Heijmans et al. (2008)
Psychological	Holocaust trauma	Human	Male and Female	Altered wake-up cortisol levels	DNA methylation (FKBP5 intron 7)	F1	Yehuda et al. (2016)
Psychological	Maternal ELA (household chaos, disorganization, emotional and physical abuse)	Human	Female (5–15 years)	Decreased infant cortisol reactivity	None reported	F1	Barclay et al. (2023)
Psychological (Socio-economic)	Economic hardships (during pregnancy)	Human	Female	Reduced birth weight; reduced head circumference	None reported	F1	Clark et al. (2021)
Physiological (Chemical)	Valproate (VPA)	Human	Male and Female	Physical malformations; neurodevelopmental disorders	Histone acetylation and methylation (based on Shafique and Winn, 2021)	F2	Martin, et al. (2022)
Physiological (Chemical)	VPA (during pregnancy)	Mouse	Female	Autistic-like behaviors; impaired sociability; increased seizure susceptibility; hyperactivity; decreased anxiety	Histone acetylation and methylation (based on Shafique and Winn, 2021)	F3	Choi et al. (2016)
Physiological (Chemical)	Bisphenol A (BPA, during pregnancy)	Mouse	Female	Obesity	Intronic DNA demethylation of Fto gene	F6	Jung et al. (2022)
Physiological (Biological)	Corticosterone	Mouse	Male: 10 weeks	Lower body weight; hyperanxiety-like behavior; altered affective behavioral responses	lncRNAs	F1–F2	Hoffmann et al. (2024)
Physiological (Biological)	Parasitic infection (Toxoplasma gondii)	Mouse	Male: 6–8 weeks	Anxiety-like; depression-like; impaired spatial working memory	miRNAs	F2	Tyebji, et al. (2020)
Physiological (Biological)	LPS (bacterial infection mimetic)	Mouse	Male: 8 weeks; Females	Heightened social interaction (F1–F2); heightened activity; only F2 had increased immune response	miRNA, tRNAs, piRNAs	F2	Liao et al. (2024)
Physiological (Biological)	PolyI:C (viral infection mimetic)	Mouse	Male	F1: depression-like behavior; altered stress response; hippocampal changes; increased immune responsivity. F2: mild behavioral changes	miRNA	F2	Kleeman et al. (2024)
Psychological	36 h light exposure; predator odor; 15 min restraint; saturated bedding	Mouse	Male: 4 weeks	Altered neurodevelopment	miRNAs	F1	Chan et al. (2020)
Psychological	Adverse childhood experiences; stress and anxiety	Human	Male: 18–25 years	Altered neurodevelopment; dysregulation in stress reactivity	miRNAs	F1	Chan et al. (2020)
Psychological	Unpredictable maternal separation and stress	Mouse	Male: PND1–14	Increased risk-taking behavior	lncRNAs	F2	Gapp et al. (2014)
Psychological	Chronic social defeat stress	Mouse	Male	Anxiety-like and depression-like symptoms (in both males and females)	lncRNAs	F1	Cunningham et al. (2021)

#### 4.3.2 Behavioral and neurodevelopmental phenotypes

Behavioral and neurodevelopmental phenotypes constitute another broad area of offspring effects due to parental exposures (Jami et al., 2021; Jawaid et al., 2021). The offspring stress response and modulation of stress response can be altered due to maternal and paternal consumption of stress hormones, environmental toxins, nutrition deficiency, and inflammation (Krishnaveni and Srinivasan, 2019; Yeramilli et al., 2023). The offspring may display increased anxiety-like and depression-like behavior; cognitive dysfunction; changes in patterns of social interactions; and problems with attention. Neurobehavioral changes have been associated with an impact on neurotransmitter systems, and these changes have been shown to be due to stress response and modulation (Bajinka et al., 2022; Harrison and Baune, 2014; Zoubovsky et al., 2022). Paternal consumption of stress, alcohol, and drug use have shown an impact on offspring brain function based on epigenetic changes within sperm. The nervous system remains highly sensitive at various developmental stages.

#### 4.3.3 Metabolic and behavioral phenotypes

Metabolic and behavioral phenotypes aside, disease susceptibility *per se* may be exaggerated in offspring as a consequence of environmental challenges imposed on parents. Congenital heart disease, hypertension, and abnormalities in cardiac morphology have been seen as a consequence of high salt diet and environmental toxins (Irakoze et al., 2021). The kidney is no less susceptible. Derangements in nephron number and glomerular filtration rate, as well as an enlarged risk of chronic kidney disease, have been noticed as a result of either intrauterine growth restriction or environmental toxins (Fukunaga and Fujita, 2023; Lorencini et al., 2025).

#### 4.3.4 Reproductive and immune system-associated phenotypes

**FIGURE 5:**
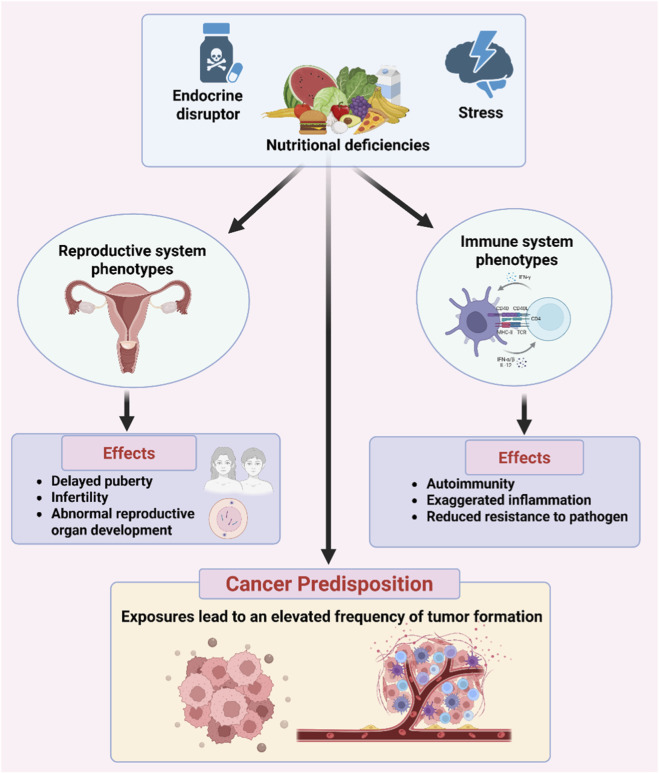
Reproductive and immune system-associated phenotypes. Environmental stressors, such as endocrine disruptors, nutrient deficiencies, and psychological stress, can alter reproductive and immune traits over time. These stressors can cause defects in gamete quality, hormone balance, and immunity, as well as epigenetic modifications, which can lead to future infertility, autoimmune diseases, and cancer susceptibility.

Reproductive and immune system-associated phenotypes are also a part of the overall disease predisposition pattern that passes through generations. It is widely recognized that endocrine-disrupting compounds, nutritional deficiencies, and stress have an inhibitory effect on gametes, impair levels of sex hormones, and disturb the developmental pattern of reproductive organs in offspring, leading to delayed puberty and infertility (Stavros et al., 2025; Zhang et al., 2024). Immune system disorders, associated with exaggerated inflammation, decreased resistance to pathogens, and an elevated predisposition toward autoimmune traits, have been associated with stress, infections, and lower maternal dietary nutrition before conception (Marques et al., 2013; Shimizu et al., 2023). Also, some exposures have shown an elevated incidence of tumor formation, indicating an association with epigenetic changes and cancer predisposition (Bouyahya et al., 2022) (Figure 5).

### 4.4 Current controversies and limitations in proving causality

Large gaps exist with regard to understanding the role of epigenetic changes caused by parents and their role in intergenerational traits. Although certain traits, some sort of behavior, and disease proneness are thought to be passed on, the principle of epigenetic marks and dependence on the germ cells for intergenerational inheritance continues to be an area of controversy. Key issues remain that, there is need for proof of successful heritability, survival and resistance to reprogramming, and maintenance at a cellular or specific tissue level. Bulk tissues form the basis for most epigenetic association studies (Fitz-James and Cavalli, 2022; Senaldi and Smith-Raska, 2020). A major issue with these technologies might be that they favor some cell types more than the rest, and resolution at a single cell level for methylation and histone marks would be more appropriate. It would be necessary to focus on controlled and bidirectional designs involving inbred strains and controlled environments. Techniques involving *in vitro* fertilization and foster transfers might also be useful.

## 5 Integration of epigenetic mechanisms in development

**FIGURE 6:**
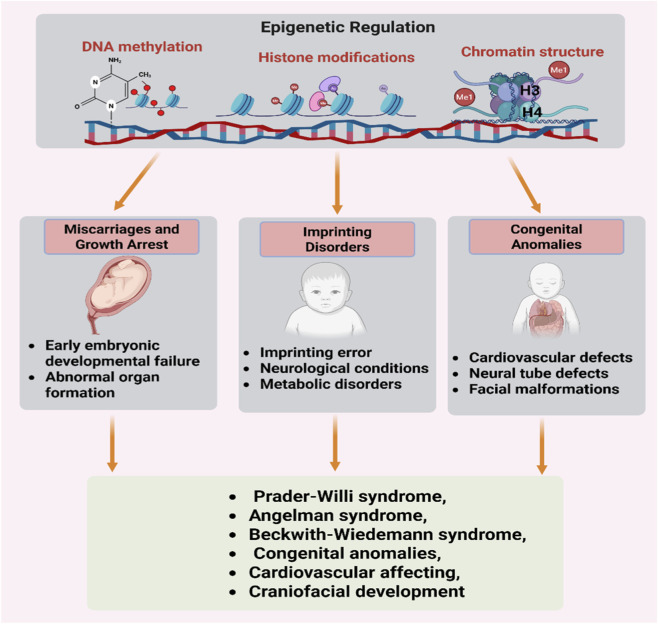
Impact of epigenetics on human health. Epigenetic regulation plays a critical role in the development and health of humans. DNA methylation, histone modifications, and chromatin structures play a critical role in the regulation of gene expression in early embryogenesis. Epigenetic regulation plays a critical role in miscarriages, growth retardation, imprinting diseases, congenital anomalies, and syndromes like Prader-Willi, Angelman, and Beckwith-Wiedemann.

These epigenetic mechanisms, including DNA methylation, histone modifications, and chromatin remodeling (Figure 6), work in concert to offer the necessary precision in space and time for embryonic development (Jang et al., 2017). In general, DNA methylation in CpG regions correlates with transcriptional repression. DNA methyl groups bind to methyl-CpG-binding proteins like MeCP2, which in turn bind to histone deacetylases (HDACs) to remove acetyl groups from histones, leading to chromatin condensation and repression of transcription (Liu et al., 2023; Liyanage et al., 2014; Miller and Grant, 2012; Rembiałkowska et al., 2025). In contrast, unmethylated DNA regions have higher levels of active histone marks like H3K4me3 (Lüscher et al., 2025; Soares et al., 2017), which indicate transcriptional activation. DNA methylation and histone modifications have been shown to operate in coherent regulatory states to activate or suppress transcription.

A new level of regulation is provided by chromatin remodeling enzymes. ATP-dependent remodelers, including SWI/SNF, ISWI, and CHD enzymes, move or remove histones to change the accessibility of DNA. Their binding is directed by histone modifications. For example, enzymes containing a bromodomain bind to acetylated histones, thus linking histone modification to accessible chromatin. This balance ensures that the effects of transient signals during development are consolidated by DNA methylation and histone modification, and active states are maintained by accessible chromatin.

For example, the HOX gene clusters are inactivated by the addition of H3K27me3 by the polycomb group complex and activated by the addition of H3K4me3 by the Trithorax group. DNA methylation maintains these states in differentiated tissues. Similarly, the pluripotency factor Oct4 (POU5F1) is associated with active chromatin in ES cells, and during differentiation, DNA methylation and histone modification stably silence its expression (Patel and Parchem, 2022; Zeineddine et al., 2014).

Epigenetic regulation strikes a balance between stability and plasticity: progressive stabilisation during differentiation maintains cell identity and specialised function (Figure 2), while high flexibility during early embryogenesis permits responsiveness to developmental and environmental cues (Ho et al., 2012; Tian and Marsit, 2018).

## 6 Impact of epigenetic on human health

Errors in epigenetic regulation in the course of early embryogenesis, including DNA methylation, histone modifications, and chromatin formation, may severely disrupt developmental processes (Rembiałkowska et al., 2025). Epigenetic regulation of key developmental genes may go awry in critical developmental stages such as pre-implantation or early organogenesis. This may result in miscarriages or growth arrest (Mu et al., 2022; Solovova and Chernykh, 2022).

Imprinted genes, which require epigenetic regulation in the form of DNA methylation patterns, may also go awry in the course of gametogenesis or embryogenesis. This may result in imprinting disorders (Gambadauro et al., 2025; Salles et al., 2025). Imprinting disorders include Prader-Willi syndrome, Angelman syndrome, and Beckwith-Wiedemann syndrome. These may present as growth retardation, neurological impairment, and metabolic problems. Epigenetic regulation in the course of embryogenesis may also result in congenital anomalies, including cardiovascular defects, craniofacial malformations, and neural tube defects (Rodríguez-Pérez et al., 2025).

Epigenetic control is closely related to early development, since even small changes in DNA methylation, histones, or chromatin composition during embryonic development can lead to a cascade of effects on cell differentiation, organogenesis, and genome integrity (Rembiałkowska et al., 2025). Knowledge in this area is important for developing prenatal diagnostics, therapy, and prevention.

Dysregulation in epigenetic control is responsible for various human diseases by affecting gene expression without any alterations in DNA sequences (Moosavi and Ardekani, 2016; Olawade et al., 2025). In estrogen receptor-positive breast cancer, drug-resistant cancer is associated with abnormal epigenetic control in DNA methylation, histones, and chromatin structure, including the reactivation of developmental gene pathways such as WNT and EMT, promoting cancer cell proliferation and metastasis (Ma et al., 2025; Feng et al., 2018; Lin et al., 2022; Macias and Hinck, 2012; Suzuki et al., 2008; Xiang et al., 2013; Serrano-Gomez et al., 2016). Similarly, loss of ARID1A alters HDAC2 levels, enhancing mitogenic signaling and suggesting potential for combined HDAC inhibitor therapies (Fukumoto et al., 2018; Xu et al., 2020).

In glioblastoma, DNA hypo- and hypermethylation, histone acetylation, and PRMT1-mediated arginine methylation lead to abnormal gene expression and tumor aggressiveness (Götze et al., 2010; Azab, 2023; Lee et al., 2015). Metabolic disorders such as type 2 diabetes are caused by abnormal DNA methylation and histone modifications in pancreatic islets, adipose tissue, and muscle, resulting in abnormal insulin secretion and inflammation (Barrès et al., 2009; Samanta et al., 2017).

Neurological disorders, including Alzheimer’s and Parkinson’s diseases, also reflect early developmental epigenetic programming, where aberrant DNA methylation and histone acetylation disrupt neurogenesis and neuronal plasticity (Yao et al., 2016; Lunnon et al., 2014; Marzi et al., 2018; Desplats et al., 2011; Soldner et al., 2016).

Collectively, these examples demonstrate that epigenetic misregulation during critical developmental windows destabilizes gene networks, linking embryonic mechanisms to lifelong disease risk and providing targets for epigenetic therapies.

## 7 Assessing the strength of the evidence and integrative epigenetic mechanisms in different developmental systems

The quality of evidence for the involvement of DNA methylation/demethylation enzymes, histone modifications, and chromatin remodelling complexes varies across study type and should be viewed in this context. There is substantial mechanistic evidence for the involvement of the core apparatus of DNA methylation, including DNMTs and TETs, as well as for the main histone modification complexes (Li et al., 2025; Modi et al., 2025). This is largely derived from controlled studies that show a direct impact on gene expression and developmental progression. Similarly, chromatin remodelling complexes such as the SWI/SNF complexes have been functionally related to nucleosome movement and transcription regulation (Li et al., 2015; Padilla-Benavides et al., 2020; Chen et al., 2024). However, the confidence in these processes varies depending upon whether they have been related to direct function, epigenomic correlation, or perturbation. While some of these mechanisms have been shown to be causal, others are more associative in nature.

Furthermore, a distinction must also be drawn between evidence derived from *in vitro* embryo studies and those derived from *in vivo* studies, as well as between evidence derived from animal studies and those derived from human studies. While *in vitro* studies, including embryo culture, are useful tools for controlling variables, they might not fully mimic the dynamic environment of early development. *In vivo* studies, especially those conducted using animal models, provide important insights into physiological relevance as well as developmental outcomes, although there might be species-specific differences (Phillips and Roth, 2019; Domínguez-Oliva et al., 2023; . Human studies provide important insights, especially from a clinical perspective, although they might be limited with respect to establishing causality.

**FIGURE 7:**
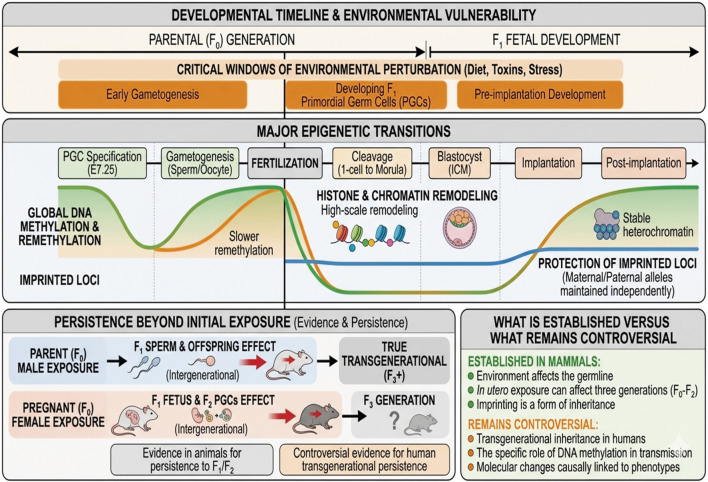
Epigenetic programming and transgenerational vulnerability timeline.

The integrative model of epigenetic regulation in development suggests that these epigenetic processes of DNA methylation, histone modification, chromatin remodeling, and three-dimensional genome organization are part of a single coordinated system that acts synergistically to regulate development (Ladel et al., 2026; Faraji and Metz, 2026). During critical developmental stages such as zygotic genome activation and implantation, there is a reprogramming of the epigenetic regulatory system that allows for the activation of embryonic gene expression (Schulz and Harrison, 2019; Sotomayor-Lugo et al., 2024). During this reprogramming, there is a global erasure of DNA methylation patterns, whereas histone modifications provide context-dependent activating or repressing cues for transcription (Lee and Surani, 2024; Pereira et al., 2024). Chromatin remodeling complexes assist in chromatin access to regulatory elements through the repositioning of histone complexes, whereas three-dimensional genome organization organizes these regulatory elements into functionally relevant domains that facilitate the regulation of gene expression. See Figure 7.

Epigenetic Programming and Transgenerational Susceptibility Timeline Figure 1: This diagram presents a conceptual model showing significant epigenetic events such as global DNA reprogramming and imprinting protection in mammalian development. This diagram emphasizes periods when environmental stressors can interfere with these processes, which may skip “erasure” events in the germline. This diagram also differentiates between FI-F2 effects and the disputed processes underlying transgenerational effects (F3).

## 8 Emerging technologies and future directions

With the help of state-of-the-art tech, epigenetics research is now turbocharged and allows researchers to focus on chromatin and gene regulation with unprecedented clarity. One of the biggest advances in recent times is single-cell epigenomics and provides insight into the epigenetic map of cells at the cellular level. Using DNA methylation or chromatin accessibility at the cellular level allows us to create maps of differences between cells in complex tissues and identify unusual cells that are often the causes of development and diseases. On the other hand, chromatin conformation capture techniques such as Hi-C enable research into three-dimensional chromatin structure, illustrating interactions between distal gene regulatory elements or gene promoters for governing gene expression. Conversely, ATAC-sequencing (Assay for Transposase-Accessible Chromatin using sequencing) yields genome-wide chromatin accessibility datasets that illustrate active regulatory potential regions or provide gene regulatory network understanding. These methodologies are increasingly linked with CRISPR-based epigenetic editing that facilitates selective alterations of DNA methylation or histone modifications targeting definite loci by creating direct evidence of gene-function relationships. Validation experiments using such methodologies make it possible to establish direct relationships between novel or changed states of gene regulation through adequate gene targeting or gene silencing techniques for understanding development principles or pathophysics of diseases with high resolution. However, irrespective of such breakthroughs, significant gaps continue to exist in the inheritance of certain gene regulation states or marks with regard to their processing or directions through the reprogramming process occurring in gametogenesis, or influence of adequate external factors or environmental influences concerning inherited gene regulation or gene regulation state transformations brought about by adequate learning or pathophysiological instruction by external forces, or their innate inheritance with regard to adequate development state influence or internalization by inheriting progenies or offsprings with regard to their adequate development state or pathophysiological applications in adequate instructions or pathophysio-logical monitoring. Such advancements enable fulfilment of translational implementation for pathophysiological monitoring with unprecedented potential for developing novel schemes for pathophysiological observations through adequate development instructions for developed schemes or monitoring devices for comprehensively understanding mysteries of development or pathophysiological transformations with regard to monitoring pathophysiological states for manipulatable or pathophysiological understanding or devising novel schemes for comprehensively understanding mysteries of adequate development or pathophysiological or monitoring transformations for understanding pathophysiological states with unprecedented development or applications.

## 9 Conclusion

In this regard, the role of molecular epigenetics in the process of embryonic development is critical. First, the proper setting and maintenance of epigenetic marks are critical for proper organogenesis. Second, aberrations in this process lead to embryonic lethality, imprinting disorders, and congenital abnormalities. These aspects highlight the importance of epigenetics in maintaining developmental homeostasis. Third, epigenetic regulation is critical for developmental plasticity and disease predisposition in subsequent generations via epigenetic inheritance. Fourth, this knowledge sheds more light on how genetics and environmental factors interact in developmental processes.

However, some important aspects in this process still pose a puzzle. These include understanding how epigenetic marks are coordinated in various developmental stages. In this regard, understanding how environmental factors during early stages of embryonic development influence epigenetic programming is critical. In this regard, understanding how such epigenetic changes are inherited in mammals is still unclear.

However, emerging technologies in this field are critical in understanding epigenetic regulation. These technologies include advances in epigenomics, chromatin conformational mapping, and CRISPR epigenome editing. These technologies will provide more insights into epigenetic regulation in various developmental processes.

## References

[B1] Adetunji A. O. Owusu H. Adewale E. F. Adesina P. A. Xedzro C. Saliu T. P. (2025). DNA methylation: a key regulator in male and female reproductive outcomes. Life 15 (7), 1109. 10.3390/life15071109 40724612 PMC12298693

[B2] Altmann S. Murani E. Schwerin M. Metges C. C. Wimmers K. Ponsuksili S. (2012). Maternal dietary protein restriction and excess affects offspring gene expression and methylation of non-SMC subunits of condensin I in liver and skeletal muscle. Epigenetics 7 (3), 239–252. 10.4161/epi.7.3.19183 22430800

[B3] Anway M. D. Cupp A. S. Uzumcu M. Skinner M. K. (2005). Epigenetic transgenerational actions of endocrine disruptors and male fertility. Science 308 (5727), 1466–1469. 10.1126/science.1108190 15933200 PMC11423801

[B4] Arand J. Reijo Pera R. A. Wossidlo M. (2021). Reprogramming of DNA methylation is linked to successful human preimplantation development. Histochem. Cell Biol. 156 (3), 197–207. 10.1007/s00418-021-02008-6 34179999 PMC8460514

[B5] Argaw-Denboba A. Schmidt T. S. Di Giacomo M. Ranjan B. Devendran S. Mastrorilli E. (2024). Paternal microbiome perturbations impact offspring fitness. Nature 629 (8012), 652–659. 10.1038/s41586-024-07336-w 38693261 PMC11096121

[B6] Ashe A. Sapetschnig A. Weick E.-M. Mitchell J. Bagijn M. P. Cording A. C. (2012). piRNAs can trigger a multigenerational epigenetic memory in the germline of C. elegans. Cell 150 (1), 88–99. 10.1016/j.cell.2012.06.018 22738725 PMC3464430

[B7] Azab M. A. (2023). The potential role of histone modifications in glioblastoma therapy. J. Mol. Pathology 4 (4), 196–212. 10.3390/jmp4040018

[B8] Azuara V. Perry P. Sauer S. Spivakov M. Jørgensen H. F. John R. M. (2006). Chromatin signatures of pluripotent cell lines. Nat. Cell Biology 8 (5), 532–538. 10.1038/ncb1403 16570078

[B9] Bajinka O. Barrow A. Mendy S. Jallow B. J. Jallow J. Barrow S. (2022). The influence of parental environmental exposure and nutrient restriction on the early life of offspring growth in Gambia—A pilot study. Int. Journal Environmental Research Public Health 19 (20), 13045. 10.3390/ijerph192013045 36293620 PMC9603272

[B10] Barclay M. E. Rinne G. R. Somers J. A. Lee S. S. Coussons-Read M. Dunkel Schetter C. (2023). Maternal early life adversity and infant stress regulation: intergenerational associations and mediation by maternal prenatal mental health. Res. Child Adolescent Psychopathology 51 (12), 1839–1855. 10.1007/s10802-022-01006-z 36508054 PMC10258218

[B11] Barrès R. Osler M. E. Yan J. Rune A. Fritz T. Caidahl K. (2009). Non-CpG methylation of the PGC-1α promoter through DNMT3B controls mitochondrial density. Cell Metab. 10 (3), 189–198. 10.1016/j.cmet.2009.07.011 19723495

[B12] Be B. Mikkelsen T. S. Xie X. Kamal M. Huebert D. J. Cuff J. (2006). A bivalent chromatin structure marks key developmental genes in embryonic stem cells. Cell 125, 315–326. 10.1016/j.cell.2006.02.041 16630819

[B13] Berghof T. Parmentier H. Lammers A. (2013). Transgenerational epigenetic effects on innate immunity in broilers: an underestimated field to be explored? Poult. Sci. 92 (11), 2904–2913. 10.3382/ps.2013-03177 24135594

[B14] Bogdarina I. Welham S. King P. J. Burns S. P. Clark A. J. (2007). Epigenetic modification of the renin-angiotensin system in the fetal programming of hypertension. Circulation Research 100 (4), 520–526. 10.1161/01.RES.0000258855.60637.58 17255528 PMC1976252

[B15] Bondarieva A. Tachibana K. (2024). Genome folding and zygotic genome activation in mammalian preimplantation embryos. Curr. Opinion Genetics and Development 89, 102268. 10.1016/j.gde.2024.102268 39383545

[B16] Bouyahya A. Mechchate H. Oumeslakht L. Zeouk I. Aboulaghras S. Balahbib A. (2022). The role of epigenetic modifications in human cancers and the use of natural compounds as epidrugs: mechanistic pathways and pharmacodynamic actions. Biomolecules 12 (3), 367. 10.3390/biom12030367 35327559 PMC8945214

[B17] Braunschweig M. Jagannathan V. Gutzwiller A. Bee G. (2012). Investigations on transgenerational epigenetic response down the male line in F2 pigs. PLoS One 7 (2), e30583. 10.1371/journal.pone.0030583 22359544 PMC3281031

[B18] Bugaut M. (1987). Occurrence, absorption and metabolism of short chain fatty acids in the digestive tract of mammals. Comp. Biochem. Physiology Part B Comp. Biochem. 86 (3), 439–472. 10.1016/0305-0491(87)90433-0 3297476

[B19] Burggren W. (2016). Epigenetic inheritance and its role in evolutionary biology: re-evaluation and new perspectives. Biology 5 (2), 24. 10.3390/biology5020024 27231949 PMC4929538

[B20] Carlini V. Policarpi C. Hackett J. A. (2022). Epigenetic inheritance is gated by naïve pluripotency and Dppa2. EMBO J. 41 (7), e108677. 10.15252/embj.2021108677 35199868 PMC8982627

[B21] Carone B. R. Fauquier L. Habib N. Shea J. M. Hart C. E. Li R. (2010). Paternally induced transgenerational environmental reprogramming of metabolic gene expression in mammals. Cell 143 (7), 1084–1096. 10.1016/j.cell.2010.12.008 21183072 PMC3039484

[B22] Chan J. C. Morgan C. P. Adrian Leu N. Shetty A. Cisse Y. M. Nugent B. M. (2020). Reproductive tract extracellular vesicles are sufficient to transmit intergenerational stress and program neurodevelopment. Nat. Communications 11 (1), 1499. 10.1038/s41467-020-15305-w 32198406 PMC7083921

[B23] Cheedipudi S. Genolet O. Dobreva G. (2014). Epigenetic inheritance of cell fates during embryonic development. Front. Genetics 5, 19. 10.3389/fgene.2014.00019 24550937 PMC3912789

[B24] Chen X. He C. Xu H. Zeng G. Huang Q. Deng Z. (2024). Characterization of the SWI/SNF complex and nucleosome organization in sorghum. Front. Plant Sci. 15, 1430467. 10.3389/fpls.2024.1430467 38988640 PMC11234113

[B25] Choi C. S. Gonzales E. L. Kim K. C. Yang S. M. Kim J.-W. Mabunga D. F. (2016). The transgenerational inheritance of autism-like phenotypes in mice exposed to valproic acid during pregnancy. Sci. Reports 6 (1), 36250. 10.1038/srep36250 27819277 PMC5098241

[B26] Clark A. E. D’Ambrosio C. Rohde N. (2021). Prenatal economic shocks and birth outcomes in UK cohort data. Econ. and Hum. Biol. 41, 100964. 10.1016/j.ehb.2020.100964 33493867

[B27] Cui Y. Deng J. Zhang Y. Du L. Jiang F. Li C. (2025). Epigenetic regulation by DNA methylation, histone modifications and chromatin remodeling complexes in controlling spermatogenesis and their dysfunction with male infertility. Cell. Mol. Life Sci. 82 (1), 343. 10.1007/s00018-025-05831-5 41051601 PMC12500515

[B28] Cunningham A. M. Walker D. M. Ramakrishnan A. Doyle M. A. Bagot R. C. Cates H. M. (2021). Sperm transcriptional state associated with paternal transmission of stress phenotypes. J. Neurosci. 41 (29), 6202–6216. 10.1523/JNEUROSCI.3192-20.2021 34099514 PMC8287983

[B29] Dabin J. Fortuny A. Polo S. E. (2016). Epigenome maintenance in response to DNA damage. Mol. Cell 62 (5), 712–727. 10.1016/j.molcel.2016.04.006 27259203 PMC5476208

[B30] Daniel Z. Brameld J. Craigon J. Scollan N. Buttery P. (2007). Effect of maternal dietary restriction during pregnancy on lamb carcass characteristics and muscle fiber composition. J. Animal Sci. 85 (6), 1565–1576. 10.2527/jas.2006-743 17296773

[B31] Dean W. Santos F. Stojkovic M. Zakhartchenko V. Walter J. Wolf E. (2001). Conservation of methylation reprogramming in mammalian development: aberrant reprogramming in cloned embryos. Proc. Natl. Acad. Sci. 98 (24), 13734–13738. 10.1073/pnas.241522698 11717434 PMC61110

[B32] Desplats P. Spencer B. Coffee E. Patel P. Michael S. Patrick C. (2011). α-Synuclein sequesters Dnmt1 from the nucleus: a novel mechanism for epigenetic alterations in lewy body diseases. J. Biological Chemistry 286 (11), 9031–9037. 10.1074/jbc.C110.212589 21296890 PMC3059002

[B33] Dias B. G. Ressler K. J. (2014). Parental olfactory experience influences behavior and neural structure in subsequent generations. Nat. Neuroscience 17 (1), 89–96. 10.1038/nn.3594 24292232 PMC3923835

[B34] Diaz-Castillo C. Chamorro-Garcia R. Shioda T. Blumberg B. (2019). Transgenerational self-reconstruction of disrupted chromatin organization after exposure to an environmental stressor in mice. Sci. Reports 9 (1), 13057. 10.1038/s41598-019-49440-2 31506492 PMC6736928

[B35] Dietz D. M. LaPlant Q. Watts E. L. Hodes G. E. Russo S. J. Feng J. (2011). Paternal transmission of stress-induced pathologies. Biol. Psychiatry 70 (5), 408–414. 10.1016/j.biopsych.2011.05.005 21679926 PMC3217197

[B36] Domínguez-Oliva A. Hernández-Ávalos I. Martínez-Burnes J. Olmos-Hernández A. Verduzco-Mendoza A. Mota-Rojas D. (2023). The importance of animal models in biomedical research: current insights and applications. Animals An Open Access Journal MDPI 13 (7), 1223. 10.3390/ani13071223 37048478 PMC10093480

[B37] Dong E. Guidotti A. Grayson D. Costa E. (2007). Histone hyperacetylation induces demethylation of reelin and 67-kDa glutamic acid decarboxylase promoters. Proc. Natl. Acad. Sci. 104 (11), 4676–4681. 10.1073/pnas.0700529104 17360583 PMC1815468

[B38] Du Z. Zhang K. Xie W. (2022). Epigenetic reprogramming in early animal development. Cold Spring Harb. Perspectives Biology 14 (6), a039677. 10.1101/cshperspect.a039677 34400552 PMC9248830

[B39] Eguizábal C. Herrera L. De Oñate L. Montserrat N. Hajkova P. Izpisúa Belmonte J. C. (2016). Characterization of the epigenetic changes during human gonadal primordial germ cells reprogramming. Stem Cells 34 (9), 2418–2428. 10.1002/stem.2422 27300161 PMC5018215

[B40] Fahey A. Brameld J. Parr T. Buttery P. (2005). The effect of maternal undernutrition before muscle differentiation on the muscle fiber development of the newborn lamb. J. Animal Sci. 83 (11), 2564–2571. 10.2527/2005.83112564x 16230653

[B41] Fallet M. Blanc M. Di Criscio M. Antczak P. Engwall M. Bosagna C. G. (2023). Present and future challenges for the investigation of transgenerational epigenetic inheritance. Environ. International 172, 107776. 10.1016/j.envint.2023.107776 36731188

[B42] Fan H. Zhang R. Tesfaye D. Tholen E. Looft C. Hölker M. (2012). Sulforaphane causes a major epigenetic repression of myostatin in porcine satellite cells. Epigenetics 7 (12), 1379–1390. 10.4161/epi.22609 23092945 PMC3528693

[B43] Fang S. Chang K.-W. Lefebvre L. (2024). Roles of endogenous retroviral elements in the establishment and maintenance of imprinted gene expression. Front. Cell Dev. Biol. 12, 1369751. 10.3389/fcell.2024.1369751 38505259 PMC10948482

[B44] Faraji J. Metz G. A. S. (2026). Environmental epigenetics: new horizons in redefining biological and health outcomes. Environ. Int. 208, 110072. 10.1016/j.envint.2026.110072 41610511

[B45] Feeney A. Nilsson E. Skinner M. K. (2014). Epigenetics and transgenerational inheritance in domesticated farm animals. J. Animal Sci. Biotechnol. 5 (1), 48. 10.1186/2049-1891-5-48 25810901 PMC4373098

[B46] Feng Y. Spezia M. Huang S. Yuan C. Zeng Z. Zhang L. (2018). Breast cancer development and progression: risk factors, cancer stem cells, signaling pathways, genomics, and molecular pathogenesis. Genes and Diseases 5 (2), 77–106. 10.1016/j.gendis.2018.05.001 30258937 PMC6147049

[B47] Fitz-James M. H. Cavalli G. (2022). Molecular mechanisms of transgenerational epigenetic inheritance. Nat. Rev. Genet. 23 (6), 325–341. 10.1038/s41576-021-00438-5 34983971 PMC7619059

[B48] Franklin T. B. Russig H. Weiss I. C. Gräff J. Linder N. Michalon A. (2010). Epigenetic transmission of the impact of early stress across generations. Biol. Psychiatry 68 (5), 408–415. 10.1016/j.biopsych.2010.05.036 20673872

[B49] Fuks F. Hurd P. J. Deplus R. Kouzarides T. (2003). The DNA methyltransferases associate with HP1 and the SUV39H1 histone methyltransferase. Nucleic Acids Research 31 (9), 2305–2312. 10.1093/nar/gkg332 12711675 PMC154218

[B50] Fukumoto T. Park P. H. Wu S. Fatkhutdinov N. Karakashev S. Nacarelli T. (2018). Repurposing Pan-HDAC inhibitors for ARID1A-mutated ovarian cancer. Cell Reports 22 (13), 3393–3400. 10.1016/j.celrep.2018.03.019 29590609 PMC5903572

[B51] Fukunaga S. Fujita Y. (2023). Low glomerular number at birth can lead to the development of chronic kidney disease. Front. Endocrinol. 14, 1120801. 10.3389/fendo.2023.1120801 36777357 PMC9909536

[B52] Fulka H. Mrazek M. Tepla O. Fulka J. (2004). DNA methylation pattern in human zygotes and developing embryos. Reproduction 128 (6), 703–708. 10.1530/rep.1.00217 15579587

[B53] Fullston T. Teague E. M. C. O. Palmer N. O. DeBlasio M. J. Mitchell M. Corbett M. (2013). Paternal obesity initiates metabolic disturbances in two generations of mice with incomplete penetrance to the F2 generation and alters the transcriptional profile of testis and sperm microRNA content. FASEB J. 27 (10), 4226–4243. 10.1096/fj.12-224048 23845863

[B54] Gambadauro A. Chirico V. Galletta F. Gulino F. Chimenz R. Serraino G. (2025). Imprinting disorders and epigenetic alterations in children conceived by assisted reproductive technologies: mechanisms, clinical outcomes, and prenatal diagnosis. Genes 16 (10), 1242. 10.3390/genes16101242 41153459 PMC12562381

[B55] Gan Q. Yoshida T. McDonald O. G. Owens G. K. (2007). Concise review: epigenetic mechanisms contribute to pluripotency and cell lineage determination of embryonic stem cells. Stem Cells 25 (1), 2–9. 10.1634/stemcells.2006-0383 17023513

[B56] Gapp K. Jawaid A. Sarkies P. Bohacek J. Pelczar P. Prados J. (2014). Implication of sperm RNAs in transgenerational inheritance of the effects of early trauma in mice. Nat. Neuroscience 17 (5), 667–669. 10.1038/nn.3695 24728267 PMC4333222

[B57] Garry F. Adams R. McCann J. Odde K. (1996). Postnatal characteristics of calves produced by nuclear transfer cloning. Theriogenology 45 (1), 141–152. 10.1016/0093-691x(95)00363-d

[B58] Gely-Pernot A. Hao C. Legoff L. Multigner L. D’Cruz S. C. Kervarrec C. (2018). Gestational exposure to chlordecone promotes transgenerational changes in the murine reproductive system of males. Sci. Reports 8 (1), 10274. 10.1038/s41598-018-28670-w 29980752 PMC6035262

[B59] Gkountela S. Li Z. Vincent J. J. Zhang K. X. Chen A. Pellegrini M. (2013). The ontogeny of cKIT+ human primordial germ cells proves to be a resource for human germ line reprogramming, imprint erasure and *in vitro* differentiation. Nat. Cell Biology 15 (1), 113–122. 10.1038/ncb2638 23242216 PMC3786872

[B60] Gonzalez-Recio O. Ugarte E. Bach A. (2012). Trans-generational effect of maternal lactation during pregnancy: a holstein cow model. PLoS One 7 (12), e51816. 10.1371/journal.pone.0051816 23284777 PMC3527476

[B61] Götze S. Wolter M. Reifenberger G. Müller O. Sievers S. (2010). Frequent promoter hypermethylation of Wnt pathway inhibitor genes in malignant astrocytic gliomas. Int. Journal Cancer 126 (11), 2584–2593. 10.1002/ijc.24981 19847810

[B62] Green B. B. Kerr D. E. (2014). Epigenetic contribution to individual variation in response to lipopolysaccharide in bovine dermal fibroblasts. Veterinary Immunol. Immunopathol. 157 (1-2), 49–58. 10.1016/j.vetimm.2013.10.015 24268632 PMC4228796

[B63] Greenberg M. V. Bourc’his D. (2019). The diverse roles of DNA methylation in mammalian development and disease. Nat. Reviews Mol. Cell Biology 20 (10), 590–607. 10.1038/s41580-019-0159-6 31399642

[B64] Gregory D. J. Kobzik L. Yang Z. McGuire C. C. Fedulov A. V. (2017). Transgenerational transmission of asthma risk after exposure to environmental particles during pregnancy. Am. J. Physiology-Lung Cell. Mol. Physiology 313 (2), L395–L405. 10.1152/ajplung.00035.2017 28495853 PMC5582941

[B65] Gu T.-P. Guo F. Yang H. Wu H.-P. Xu G.-F. Liu W. (2011). The role of Tet3 DNA dioxygenase in epigenetic reprogramming by oocytes. Nature 477 (7366), 606–610. 10.1038/nature10443 21892189

[B66] Gujar H. Weisenberger D. J. Liang G. (2019). The roles of human DNA methyltransferases and their isoforms in shaping the epigenome. Genes 10 (2), 172. 10.3390/genes10020172 30813436 PMC6409524

[B67] Guo X. Yang J. (2024). Advances in DNA methylation of imprinted genes and folic acid regulation of growth and development. Epigenomics 16 (15-16), 1117–1127. 10.1080/17501911.2024.2384833 39140401 PMC11418287

[B68] Guo H. Zhu P. Yan L. Li R. Hu B. Lian Y. (2014). The DNA methylation landscape of human early embryos. Nature 511 (7511), 606–610. 10.1038/nature13544 25079557

[B69] Hackett J. A. Surani M. A. (2014). Regulatory principles of pluripotency: from the ground state up. Cell Stem Cell 15 (4), 416–430. 10.1016/j.stem.2014.09.015 25280218

[B70] Hanna C. W. Peñaherrera M. S. Saadeh H. Andrews S. McFadden D. E. Kelsey G. (2016). Pervasive polymorphic imprinted methylation in the human placenta. Genome Research 26 (6), 756–767. 10.1101/gr.196139.115 26769960 PMC4889973

[B71] Hao C. Gely-Pernot A. Kervarrec C. Boudjema M. Becker E. Khil P. (2016). Exposure to the widely used herbicide atrazine results in deregulation of global tissue-specific RNA transcription in the third generation and is associated with a global decrease of histone trimethylation in mice. Nucleic Acids Research 44 (20), 9784–9802. 10.1093/nar/gkw840 27655631 PMC5175363

[B72] Hardikar A. A. Satoor S. N. Karandikar M. S. Joglekar M. V. Puranik A. S. Wong W. (2015). Multigenerational undernutrition increases susceptibility to obesity and diabetes that is not reversed after dietary recuperation. Cell Metab. 22 (2), 312–319. 10.1016/j.cmet.2015.06.008 26166746

[B73] Harrison E. Baune B. (2014). Modulation of early stress-induced neurobiological changes: a review of behavioural and pharmacological interventions in animal models. Transl. Psychiatry 4 (5), e390. 10.1038/tp.2014.31 24825729 PMC4035722

[B74] Heijmans B. T. Tobi E. W. Stein A. D. Putter H. Blauw G. J. Susser E. S. (2008). Persistent epigenetic differences associated with prenatal exposure to famine in humans. Proc. Natl. Acad. Sci. 105 (44), 17046–17049. 10.1073/pnas.0806560105 18955703 PMC2579375

[B75] Hemberger M. Dean W. Reik W. (2009). Epigenetic dynamics of stem cells and cell lineage commitment: digging Waddington's canal. Nat. Reviews Mol. Cell Biology 10 (8), 526–537. 10.1038/nrm2727 19603040

[B76] Ho L. Crabtree G. R. (2010). Chromatin remodelling during development. Nature 463 (7280), 474–484. 10.1038/nature08911 20110991 PMC3060774

[B77] Ho S.-M. Johnson A. Tarapore P. Janakiram V. Zhang X. Leung Y.-K. (2012). Environmental epigenetics and its implication on disease risk and health outcomes. ILAR Journal 53 (3-4), 289–305. 10.1093/ilar.53.3-4.289 23744968 PMC4021822

[B78] Hodawadekar S. Marmorstein R. (2007). Chemistry of acetyl transfer by histone modifying enzymes: structure, mechanism and implications for effector design. Oncogene 26 (37), 5528–5540. 10.1038/sj.onc.1210619 17694092

[B79] Hoffmann L. Li B. Zhao Q. Wei W. Leighton L. Bredy T. (2024). Chronically high stress hormone levels dysregulate sperm long noncoding RNAs and their embryonic microinjection alters development and affective behaviours. Mol. Psychiatry 29 (3), 590–601. 10.1038/s41380-023-02350-2 38114632

[B80] Holuka C. Grova N. Charalambous E. G. Le CléacH J. Turner J. D. Mposhi A. (2024). Transgenerational impacts of early life adversity: from health determinants, implications to epigenetic consequences. Neurosci. and Biobehav. Rev. 164, 105785. 10.1016/j.neubiorev.2024.105785 38945418

[B81] Hu Y. Xu H. Li Z. Zheng X. Jia X. Nie Q. (2013). Comparison of the genome-wide DNA methylation profiles between fast-growing and slow-growing broilers. PLoS One 8 (2), e56411. 10.1371/journal.pone.0056411 23441189 PMC3575439

[B82] Huang M.-B. Xu H. Xie S.-J. Zhou H. Qu L.-H. (2011). Insulin-like growth factor-1 receptor is regulated by microRNA-133 during skeletal myogenesis. PLoS One 6 (12), e29173. 10.1371/journal.pone.0029173 22195016 PMC3240640

[B83] Huang Y. Liu H. Du H. Zhang W. Kang X. Luo Y. (2019). Developmental features of DNA methylation in CpG islands of human gametes and preimplantation embryos. Exp. Ther. Med. 17 (6), 4447–4456. 10.3892/etm.2019.7523 31105782 PMC6507515

[B84] Huang Y. Guo J. He X.-J. Li C. (2025). Chromatin remodeling in plants: complex composition, mechanistic diversity, and biological functions. Mol. Plant 18 (9), 1436–1457. 10.1016/j.molp.2025.08.004 40808254

[B85] Huang Z. Hu L. Liu Z. Wang S. (2025). The functions and regulatory mechanisms of histone modifications in skeletal muscle development and disease. Int. J. Mol. Sci. 26 (8), 3644. 10.3390/ijms26083644 40332229 PMC12027200

[B86] Huypens P. Sass S. Wu M. Dyckhoff D. Tschöp M. Theis F. (2016). Epigenetic germline inheritance of diet-induced obesity and insulin resistance. Nat. Genetics 48 (5), 497–499. 10.1038/ng.3527 26974008

[B87] Igbayilola Y. Morakinyo A. Iranloye B. (2021). Adverse effects of perinatal protein restriction on glucose homeostasis in offspring of Sprague-dawley rats. Sci. Afr. 14, e01036. 10.1016/j.sciaf.2021.e01036

[B88] Irakoze L. Manirakiza A. Zhang Y. Liu J. Li J. Nkengurutse L. (2021). Metabolic syndrome in offspring of parents with metabolic syndrome: a meta-analysis. Obes. Facts 14 (1), 148–162. 10.1159/000513370 33508842 PMC7983676

[B89] Irie N. Weinberger L. Tang W. W. Kobayashi T. Viukov S. Manor Y. S. (2015). SOX17 is a critical specifier of human primordial germ cell fate. Cell 160 (1), 253–268. 10.1016/j.cell.2014.12.013 25543152 PMC4310934

[B90] Ishida M. Moore G. E. (2013). The role of imprinted genes in humans. Mol. Aspects Medicine 34 (4), 826–840. 10.1016/j.mam.2012.06.009 22771538

[B91] Ivanova E. Canovas S. Garcia-Martínez S. Romar R. Lopes J. S. Rizos D. (2020). DNA methylation changes during preimplantation development reveal inter-species differences and reprogramming events at imprinted genes. Clin. Epigenetics 12 (1), 64. 10.1186/s13148-020-00857-x 32393379 PMC7216732

[B92] Jami E. S. Hammerschlag A. R. Bartels M. Middeldorp C. M. (2021). Parental characteristics and offspring mental health and related outcomes: a systematic review of genetically informative literature. Transl. Psychiatry 11 (1), 197. 10.1038/s41398-021-01300-2 33795643 PMC8016911

[B93] Jang H. S. Shin W. J. Lee J. E. Do J. T. (2017). CpG and non-CpG methylation in epigenetic gene regulation and brain function. Genes 8 (6), 148. 10.3390/genes8060148 28545252 PMC5485512

[B94] Jawaid A. Jehle K.-L. Mansuy I. M. (2021). Impact of parental exposure on offspring health in humans. Trends Genet. 37 (4), 373–388. 10.1016/j.tig.2020.10.006 33189388

[B95] Jung Y. H. Wang H.-L. V. Ruiz D. Bixler B. J. Linsenbaum H. Xiang J.-F. (2022). Recruitment of CTCF to an Fto enhancer is responsible for transgenerational inheritance of BPA-induced obesity. Proc. Natl. Acad. Sci. 119 (50), e2214988119. 10.1073/pnas.2214988119 36469784 PMC9897486

[B96] Kakoulidou I. Avramidou E. V. Baránek M. Brunel-Muguet S. Farrona S. Johannes F. (2021). Epigenetics for crop improvement in times of global change. Biology 10 (8), 766. 10.3390/biology10080766 34439998 PMC8389687

[B97] Kleeman E. A. Reisinger S. N. Adithya P. Houston B. Stathatos G. Garnham A. L. (2024). Paternal immune activation by poly I: c modulates sperm noncoding RNA profiles and causes transgenerational changes in offspring behavior. Brain, Behav. Immun. 115, 258–279. 10.1016/j.bbi.2023.10.005 37820975

[B98] Krishnaveni G. V. Srinivasan K. (2019). Maternal nutrition and offspring stress response—Implications for future development of non-communicable disease: a perspective from India. Front. Psychiatry 10, 795. 10.3389/fpsyt.2019.00795 31736810 PMC6829676

[B99] Kruip T. A. Den Daas J. (1997). *In vitro* produced and cloned embryos: effects on pregnancy, parturition and offspring. Theriogenology 47 (1), 43–52. 10.1016/s0093-691x(96)00338-x

[B100] Ladel L. Sailo B. Das P. Lin E. S. Tan W. Y. Chhoda A. (2026). Epigenetic regulation of higher-order chromatin structure (HOCS) and its implication in human diseases. Cancers 18 (3), 483. 10.3390/cancers18030483 41681953 PMC12897372

[B101] Lan F. Shi Y. (2009). Epigenetic regulation: methylation of histone and non-histone proteins. Sci. China Ser. C Life Sci. 52 (4), 311–322. 10.1007/s11427-009-0054-z 19381457

[B102] Lee S. M. Surani M. A. (2024). Epigenetic reprogramming in mouse and human primordial germ cells. Exp. and Molecular Medicine 56 (12), 2578–2587. 10.1038/s12276-024-01359-z 39672813 PMC11671582

[B103] Lee P. Murphy B. Miller R. Menon V. Banik N. L. Giglio P. (2015). Mechanisms and clinical significance of histone deacetylase inhibitors: epigenetic glioblastoma therapy. Anticancer Research 35 (2), 615–625. 25667438 PMC6052863

[B104] Li R. W. Li C. (2006). Butyrate induces profound changes in gene expression related to multiple signal pathways in bovine kidney epithelial cells. BMC Genomics 7 (1), 234. 10.1186/1471-2164-7-234 16972989 PMC1592091

[B105] Li M. Hada A. Sen P. Olufemi L. Hall M. A. Smith B. Y. (2015). Dynamic regulation of transcription factors by nucleosome remodeling. eLife 4, e06249. 10.7554/eLife.06249 26047462 PMC4456607

[B106] Li G. Yu Y. Fan Y. Li C. Xu X. Duan J. (2017). Genome wide abnormal DNA methylome of human blastocyst in assisted reproductive technology. J. Genet. Genomics 44 (10), 475–481. 10.1016/j.jgg.2017.09.001 29037989

[B107] Li J. Liao Q. Guo Y. Zhang J. Zhang R. Liu Q. (2025). Mechanism of crosstalk between DNA methylation and histone acetylation and related advances in diagnosis and treatment of premature ovarian failure. Epigenetics 20 (1), 2528563. 10.1080/15592294.2025.2528563 40620015 PMC12239803

[B108] Liao H. Lu D. Reisinger S. N. Kleeman E. A. van de Garde N. Gubert C. (2024). Mimicking bacterial infection in male mice changes sperm small RNA profiles and multigenerationally alters offspring behavior and physiology. Brain, Behav. Immun. 119, 520–538. 10.1016/j.bbi.2024.04.017 38636562

[B109] Lin K. Baritaki S. Vivarelli S. Falzone L. Scalisi A. Libra M. (2022). The breast cancer protooncogenes HER2, BRCA1 and BRCA2 and their regulation by the iNOS/NOS2 axis. Antioxidants 11 (6), 1195. 10.3390/antiox11061195 35740092 PMC9227079

[B110] Liu X. Wang J. Li R. Yang X. Sun Q. Albrecht E. (2011). Maternal dietary protein affects transcriptional regulation of myostatin gene distinctively at weaning and finishing stages in skeletal muscle of Meishan pigs. Epigenetics 6 (7), 899–907. 10.4161/epi.6.7.16005 21597337

[B111] Liu R. Zhao E. Yu H. Yuan C. Abbas M. N. Cui H. (2023). Methylation across the central dogma in health and diseases: new therapeutic strategies. Signal Transduct. Target. Ther. 8 (1), 310. 10.1038/s41392-023-01528-y 37620312 PMC10449936

[B112] Liyanage V. R. Jarmasz J. S. Murugeshan N. Del Bigio M. R. Rastegar M. Davie J. R. (2014). DNA modifications: function and applications in normal and disease states. Biology 3 (4), 670–723. 10.3390/biology3040670 25340699 PMC4280507

[B113] Lorencini D. A. Lopes P. C. Bettiol H. Barbieri M. A. Coelho E. B. (2025). Association between intrauterine growth restriction and chronic kidney disease: a birth cohort study analysis. Kidney Med. 7, 101120. 10.1016/j.xkme.2025.101120 41209156 PMC12595368

[B114] Lunnon K. Smith R. Hannon E. De Jager P. L. Srivastava G. Volta M. (2014). Methylomic profiling implicates cortical deregulation of ANK1 in alzheimer's disease. Nat. Neuroscience 17 (9), 1164–1170. 10.1038/nn.3782 25129077 PMC4410018

[B115] Luo C. Hajkova P. Ecker J. R. (2018). Dynamic DNA methylation: in the right place at the right time. Science 361 (6409), 1336–1340. 10.1126/science.aat6806 30262495 PMC6197482

[B116] Lüscher B. Bussmann P. Müller J. (2025). Role of histone H3 lysine 4 methylation in chromatin biology. Molecules 30 (20), 4075. 10.3390/molecules30204075 41157092 PMC12565824

[B117] Ma C. Cheng J. Gu J. Wang Q. (2025). Epigenetic drugs in cancer therapy: mechanisms, immune modulation, and therapeutic applications. Mol. Biomed. 6 (1), 132. 10.1186/s43556-025-00373-5 41335282 PMC12675902

[B118] Macias H. Hinck L. (2012). Mammary gland development. Wiley Interdiscip. Rev. Dev. Biol. 1 (4), 533–557. 10.1002/wdev.35 22844349 PMC3404495

[B119] Magnúsdóttir E. Dietmann S. Murakami K. Günesdogan U. Tang F. Bao S. (2013). A tripartite transcription factor network regulates primordial germ cell specification in mice. Nat. Cell Biology 15 (8), 905–915. 10.1038/ncb2798 23851488 PMC3796875

[B120] Manikkam M. Tracey R. Guerrero-Bosagna C. Skinner M. K. (2012). Pesticide and insect repellent mixture (permethrin and DEET) induces epigenetic transgenerational inheritance of disease and sperm epimutations. Reprod. Toxicology 34 (4), 708–719. 10.1016/j.reprotox.2012.08.010 22975477 PMC3513590

[B121] Manikkam M. Tracey R. Guerrero-Bosagna C. Skinner M. K. (2013). Plastics derived endocrine disruptors (BPA, DEHP and DBP) induce epigenetic transgenerational inheritance of obesity, reproductive disease and sperm epimutations. PLoS One 8 (1), e55387. 10.1371/journal.pone.0055387 23359474 PMC3554682

[B122] Manikkam M. Haque M. M. Guerrero-Bosagna C. Nilsson E. E. Skinner M. K. (2014). Pesticide methoxychlor promotes the epigenetic transgenerational inheritance of adult-onset disease through the female germline. PLoS One 9 (7), e102091. 10.1371/journal.pone.0102091 25057798 PMC4109920

[B123] Margueron R. Reinberg D. (2011). The polycomb complex PRC2 and its mark in life. Nature 469 (7330), 343–349. 10.1038/nature09784 21248841 PMC3760771

[B124] Marques A. H. O'Connor T. G. Roth C. Susser E. Bjørke-Monsen A.-L. (2013). The influence of maternal prenatal and early childhood nutrition and maternal prenatal stress on offspring immune system development and neurodevelopmental disorders. Front. Neuroscience 7, 120. 10.3389/fnins.2013.00120 PMC3728489 23914151

[B125] Martin M. Hill C. Bewley S. MacLennan A. H. Braillon A. (2022). Transgenerational adverse effects of valproate? A patient report from 90 affected families. Birth Defects Res. 114 (1), 13–16. 10.1002/bdr2.1967 34866359

[B126] Marzi S. J. Leung S. K. Ribarska T. Hannon E. Smith A. R. Pishva E. (2018). A histone acetylome-wide association study of Alzheimer’s disease identifies disease-associated H3K27ac differences in the entorhinal cortex. Nat. Neuroscience 21 (11), 1618–1627. 10.1038/s41593-018-0253-7 30349106

[B127] Masuyama H. Mitsui T. Eguchi T. Tamada S. Hiramatsu Y. (2016). The effects of paternal high-fat diet exposure on offspring metabolism with epigenetic changes in the mouse adiponectin and leptin gene promoters. Am. J. Physiology-Endocrinology Metabolism 311 (1), E236–E245. 10.1152/ajpendo.00095.2016 27245335

[B128] McBirney M. King S. E. Pappalardo M. Houser E. Unkefer M. Nilsson E. (2017). Atrazine induced epigenetic transgenerational inheritance of disease, lean phenotype and sperm epimutation pathology biomarkers. PLoS One 12 (9), e0184306. 10.1371/journal.pone.0184306 28931070 PMC5606923

[B129] Md N. (2002). Diet, methyl donors and DNA methylation: interactions between dietary folate, methionine and choline. J. Nutr. 132, 2333S–2335S. 12163687 10.1093/jn/132.8.2333S

[B130] Miller J. L. Grant P. A. (2012). The role of DNA methylation and histone modifications in transcriptional regulation in humans. Epigenetics Dev. Dis. 61, 289–317. 10.1007/978-94-007-4525-4_13 23150256 PMC6611551

[B131] Modi N. Guo J. Lee R. A. Greenstein A. Lee R. S. (2025). Targeted DNA methylation using modified DNA probes: a potential therapeutic tool for depression and stress-related disorders. Int. J. Mol. Sci. 26 (12), 5643. 10.3390/ijms26125643 40565107 PMC12193527

[B132] Moosavi A. Ardekani A. M. (2016). Role of epigenetics in biology and human diseases. Iran. Biomedical Journal 20 (5), 246–258. 10.22045/ibj.2016.01 27377127 PMC5075137

[B133] Mu J. Zhou Z. Sang Q. Wang L. (2022). The physiological and pathological mechanisms of early embryonic development. Fundam. Res. 2 (6), 859–872. 10.1016/j.fmre.2022.08.011 38933386 PMC11197659

[B134] Nan X. Ng H.-H. Johnson C. A. Laherty C. D. Turner B. M. Eisenman R. N. (1998). Transcriptional repression by the methyl-CpG-binding protein MeCP2 involves a histone deacetylase complex. Nature 393 (6683), 386–389. 10.1038/30764 9620804

[B135] Ng S.-F. Lin R. C. Laybutt D. R. Barres R. Owens J. A. Morris M. J. (2010). Chronic high-fat diet in fathers programs β-cell dysfunction in female rat offspring. Nature 467 (7318), 963–966. 10.1038/nature09491 20962845

[B136] Nowacka-Woszuk J. Szczerbal I. Malinowska A. M. Chmurzynska A. (2018). Transgenerational effects of prenatal restricted diet on gene expression and histone modifications in the rat. PLoS One 13 (2), e0193464. 10.1371/journal.pone.0193464 29474484 PMC5825138

[B137] Oksuz O. Henninger J. E. Warneford-Thomson R. Zheng M. M. Erb H. Vancura A. (2023). Transcription factors interact with RNA to regulate genes. Mol. Cell 83 (14), 2449–2463. e2413. 10.1016/j.molcel.2023.06.012 37402367 PMC10529847

[B138] Olawade D. B. Rashad I. Egbon E. Teke J. Ovsepian S. V. Boussios S. (2025). Reversing epigenetic dysregulation in neurodegenerative diseases: mechanistic and therapeutic considerations. Int. J. Mol. Sci. 26 (10), 4929. 10.3390/ijms26104929 40430067 PMC12112518

[B139] Ou J.-N. Torrisani J. Unterberger A. Provençal N. Shikimi K. Karimi M. (2007). Histone deacetylase inhibitor trichostatin A induces global and gene-specific DNA demethylation in human cancer cell lines. Biochem. Pharmacology 73 (9), 1297–1307. 10.1016/j.bcp.2006.12.032 17276411

[B140] Padilla-Benavides T. Reyes-Gutierrez P. Imbalzano A. N. (2020). Regulation of the mammalian SWI/SNF family of chromatin remodeling enzymes by phosphorylation during myogenesis. Biology 9 (7), 152. 10.3390/biology9070152 32635263 PMC7407365

[B141] Patel I. Parchem R. J. (2022). Regulation of Oct4 in stem cells and neural crest cells. Birth Defects Res. 114 (16), 983–1002. 10.1002/bdr2.2007 35365980 PMC9525453

[B142] Pereira B. Correia F. P. Alves I. A. Costa M. Gameiro M. Martins A. P. (2024). Epigenetic reprogramming as a key to reverse ageing and increase longevity. Ageing Res. Rev. 95, 102204. 10.1016/j.arr.2024.102204 38272265

[B143] Petrussa L. Van de Velde H. De Rycke M. (2016). Similar kinetics for 5‐methylcytosine and 5‐hydroxymethylcytosine during human preimplantation development *in vitro* . Mol. Reproduction Development 83 (7), 594–605. 10.1002/mrd.22656 27163211

[B144] Phillips N. L. H. Roth T. L. (2019). Animal models and their contribution to our understanding of the relationship between environments, epigenetic modifications, and behavior. Genes 10 (1), 47. 10.3390/genes10010047 30650619 PMC6357183

[B145] Pistek V. L. Fürst R. W. Kliem H. Bauersachs S. Meyer H. H. Ulbrich S. E. (2013). HOXA10 mRNA expression and promoter DNA methylation in female pig offspring after in utero estradiol-17β exposure. J. Steroid Biochemistry Molecular Biology 138, 435–444. 10.1016/j.jsbmb.2013.09.006 24056088

[B146] Platenburg G. J. Vollebregt E. J. Karatzas C. N. Kootwijk E. P. De Boer H. A. Strijker R. (1996). Mammary gland-specific hypomethylation of Hpa II sites flanking the bovine αS1-casein gene. Transgenic Research 5 (6), 421–431. 10.1007/BF01980207 8840525

[B147] Prajapati H. K. Ocampo J. Clark D. J. (2020). Interplay among ATP-dependent chromatin remodelers determines chromatin organisation in yeast. Biology 9 (8), 190. 10.3390/biology9080190 32722483 PMC7466152

[B148] Quarato P. Singh M. Bourdon L. Cecere G. (2022). Inheritance and maintenance of small RNA‐mediated epigenetic effects. Bioessays 44 (6), 2100284. 10.1002/bies.202100284 35338497

[B149] Rando O. J. (2012). Daddy issues: paternal effects on phenotype. Cell 151 (4), 702–708. 10.1016/j.cell.2012.10.020 23141533 PMC3564497

[B150] Rembiałkowska N. Rekiel K. Urbanowicz P. Mamala M. Marczuk K. Wojtaszek M. (2025). Epigenetic dysregulation in cancer: implications for gene expression and DNA repair-associated pathways. Int. J. Mol. Sci. 26 (13), 6531. 10.3390/ijms26136531 40650308 PMC12249909

[B151] Ren J. Cheng Y. Ming Z.-H. Dong X.-Y. Zhou Y.-Z. Ding G.-L. (2018). Intrauterine hyperglycemia exposure results in intergenerational inheritance *via* DNA methylation reprogramming on F1 PGCs. Epigenetics and Chromatin 11 (1), 20. 10.1186/s13072-018-0192-2 29801514 PMC5968593

[B152] Rodgers A. B. Morgan C. P. Bronson S. L. Revello S. Bale T. L. (2013). Paternal stress exposure alters sperm microRNA content and reprograms offspring HPA stress axis regulation. J. Neurosci. 33 (21), 9003–9012. 10.1523/JNEUROSCI.0914-13.2013 23699511 PMC3712504

[B153] Rodríguez-Pérez J. M. Ortega-Zhindón D. B. Villamil-Castañeda C. Lara-Ortiz J. S. Borgonio-Cuadra V. M. Cervantes-Salazar J. L. (2025). Congenital heart diseases: recent insights into epigenetic mechanisms. Cells 14 (11), 820. 10.3390/cells14110820 40497996 PMC12154987

[B154] Salles J. Gorse T. Benvegnu G. Raynaud J.-P. Tauber M. (2025). Imprinting disorders as a window to understand pediatric feeding disorders. Orphanet J. Rare Dis. 20 (1), 247. 10.1186/s13023-025-03789-y 40413507 PMC12102808

[B155] Samanta S. Rajasingh S. Cao T. Dawn B. Rajasingh J. (2017). Epigenetic dysfunctional diseases and therapy for infection and inflammation. Biochimica Biophysica Acta (BBA)-Molecular Basis Dis. 1863 (2), 518–528. 10.1016/j.bbadis.2016.11.030 27919711 PMC5222695

[B156] Sanchez-Delgado M. Court F. Vidal E. Medrano J. Monteagudo-Sánchez A. Martin-Trujillo A. (2016). Human oocyte-derived methylation differences persist in the placenta revealing widespread transient imprinting. PLoS Genetics 12 (11), e1006427. 10.1371/journal.pgen.1006427 27835649 PMC5106035

[B157] Schneider S. Marxfeld H. Gröters S. Buesen R. van Ravenzwaay B. (2013). Vinclozolin—no transgenerational inheritance of anti-androgenic effects after maternal exposure during organogenesis via the intraperitoneal route. Reprod. Toxicology 37, 6–14. 10.1016/j.reprotox.2012.12.003 23313085

[B158] Schulz K. N. Harrison M. M. (2019). Mechanisms regulating zygotic genome activation. Nat. Reviews. Genet. 20 (4), 221–234. 10.1038/s41576-018-0087-x 30573849 PMC6558659

[B159] Senaldi L. Smith-Raska M. (2020). Evidence for germline non-genetic inheritance of human phenotypes and diseases. Clin. Epigenetics 12 (1), 136. 10.1186/s13148-020-00929-y 32917273 PMC7488552

[B160] Serrano-Gomez S. J. Maziveyi M. Alahari S. K. (2016). Regulation of epithelial-mesenchymal transition through epigenetic and post-translational modifications. Mol. Cancer 15 (1), 18. 10.1186/s12943-016-0502-x 26905733 PMC4765192

[B161] Shimizu Y. Sakata-Haga H. Saikawa Y. Hatta T. (2023). Influence of immune system abnormalities caused by maternal immune activation in the postnatal period. Cells 12 (5), 741. 10.3390/cells12050741 36899877 PMC10001371

[B162] Sinclair K. D. Allegrucci C. Singh R. Gardner D. S. Sebastian S. Bispham J. (2007). DNA methylation, insulin resistance, and blood pressure in offspring determined by maternal periconceptional B vitamin and methionine status. Proc. Natl. Acad. Sci. 104 (49), 19351–19356. 10.1073/pnas.0707258104 18042717 PMC2148293

[B163] Singh S. P. Chand H. S. Langley R. J. Mishra N. Barrett T. Rudolph K. (2017). Gestational exposure to sidestream (secondhand) cigarette smoke promotes transgenerational epigenetic transmission of exacerbated allergic asthma and bronchopulmonary dysplasia. J. Immunol. 198 (10), 3815–3822. 10.4049/jimmunol.1700014 28381639 PMC5473031

[B164] Singh A. Rappolee D. A. Ruden D. M. (2023). Epigenetic reprogramming in mice and humans: from fertilization to primordial germ cell development. Cells 12 (14), 1874. 10.3390/cells12141874 37508536 PMC10377882

[B165] Skinner M. K. Anway M. D. Savenkova M. I. Gore A. C. Crews D. (2008). Transgenerational epigenetic programming of the brain transcriptome and anxiety behavior. PLoS One 3 (11), e3745. 10.1371/journal.pone.0003745 19015723 PMC2581440

[B166] Skinner M. K. Manikkam M. Tracey R. Guerrero-Bosagna C. Haque M. Nilsson E. E. (2013). Ancestral dichlorodiphenyltrichloroethane (DDT) exposure promotes epigenetic transgenerational inheritance of obesity. BMC Medicine 11 (1), 228. 10.1186/1741-7015-11-228 24228800 PMC3853586

[B167] Smith Z. D. Chan M. M. Humm K. C. Karnik R. Mekhoubad S. Regev A. (2014). DNA methylation dynamics of the human preimplantation embryo. Nature 511 (7511), 611–615. 10.1038/nature13581 25079558 PMC4178976

[B168] Soares L. M. He P. C. Chun Y. Suh H. Kim T. Buratowski S. (2017). Determinants of histone H3K4 methylation patterns. Mol. Cell 68 (4), 773–785. e776. 10.1016/j.molcel.2017.10.013 29129639 PMC5706784

[B169] Soldner F. Stelzer Y. Shivalila C. S. Abraham B. J. Latourelle J. C. Barrasa M. I. (2016). Parkinson-associated risk variant in distal enhancer of α-synuclein modulates target gene expression. Nature 533 (7601), 95–99. 10.1038/nature17939 27096366 PMC5042324

[B170] Solovova O. A. Chernykh V. B. (2022). Genetics of oocyte maturation defects and early embryo development arrest. Genes 13 (11), 1920. 10.3390/genes13111920 36360157 PMC9689903

[B171] Sotomayor-Lugo F. Iglesias-Barrameda N. Castillo-Aleman Y. M. Casado-Hernandez I. Villegas-Valverde C. A. Bencomo-Hernandez A. A. (2024). The dynamics of histone modifications during mammalian zygotic genome activation. Int. J. Mol. Sci. 25 (3), 1459. 10.3390/ijms25031459 38338738 PMC10855761

[B172] Soubry A. (2015). Epigenetic inheritance and evolution: a paternal perspective on dietary influences. Prog. Biophysics Molecular Biology 118 (1-2), 79–85. 10.1016/j.pbiomolbio.2015.02.008 25769497

[B173] Stavros S. Kathopoulis N. Moustakli E. Potiris A. Anagnostaki I. Topis S. (2025). Endocrine-disrupting chemicals and Male infertility: mechanisms, risks, and regulatory challenges. J. Xenobiotics 15 (5), 165. 10.3390/jox15050165 41149751 PMC12565704

[B174] Stouder C. Paoloni-Giacobino A. (2010). Transgenerational effects of the endocrine disruptor vinclozolin on the methylation pattern of imprinted genes in the mouse sperm. Reproduction 139 (2), 373–379. 10.1530/REP-09-0340 19887539

[B175] Suganuma T. Workman J. L. (2011). Signals and combinatorial functions of histone modifications. Annu. Review Biochemistry 80 (1), 473–499. 10.1146/annurev-biochem-061809-175347 21529160

[B176] Suzuki H. Toyota M. Caraway H. Gabrielson E. Ohmura T. Fujikane T. (2008). Frequent epigenetic inactivation of wnt antagonist genes in breast cancer. Br. Journal Cancer 98 (6), 1147–1156. 10.1038/sj.bjc.6604259 PMC2275475 18283316

[B177] Swanson K. Stelwagen K. Dobson J. Henderson H. Davis S. Farr V. (2009). Transcriptome profiling of streptococcus uberis-induced mastitis reveals fundamental differences between immune gene expression in the mammary gland and in a primary cell culture model. J. Dairy Science 92 (1), 117–129. 10.3168/jds.2008-1382 19109270

[B178] Takahashi N. Coluccio A. Thorball C. W. Planet E. Shi H. Offner S. (2019). ZNF445 is a primary regulator of genomic imprinting. Genes and Development 33 (1-2), 49–54. 10.1101/gad.320069.118 30602440 PMC6317318

[B179] Tarleton B. J. Wiley A. A. Bartol F. F. (2001). Neonatal estradiol exposure alters uterine morphology and endometrial transcriptional activity in prepubertal gilts. Domest. Anim. Endocrinol. 21 (2), 111–125. 10.1016/s0739-7240(01)00106-0 11585701

[B180] Tian F.-Y. Marsit C. J. (2018). Environmentally induced epigenetic plasticity in development: epigenetic toxicity and epigenetic adaptation. Curr. Epidemiology Reports 5 (4), 450–460. 10.1007/s40471-018-0175-7 30984515 PMC6456900

[B181] Tian F. Zhan F. VanderKraats N. D. Hiken J. F. Edwards J. R. Zhang H. (2013). DNMT gene expression and methylome in Marek’s disease resistant and susceptible chickens prior to and following infection by MDV. Epigenetics 8 (4), 431–444. 10.4161/epi.24361 23538681 PMC3674052

[B182] Tompkins J. D. (2023). Transgenerational epigenetic DNA methylation editing and human disease. Biomolecules 13 (12), 1684. 10.3390/biom13121684 38136557 PMC10742326

[B183] Torres-Garcia S. Yaseen I. Shukla M. Audergon P. N. White S. A. Pidoux A. L. (2020). Epigenetic gene silencing by heterochromatin primes fungal resistance. Nature 585 (7825), 453–458. 10.1038/s41586-020-2706-x 32908306 PMC7116710

[B184] Tracey R. Manikkam M. Guerrero-Bosagna C. Skinner M. K. (2013). Hydrocarbons (jet fuel JP-8) induce epigenetic transgenerational inheritance of obesity, reproductive disease and sperm epimutations. Reprod. Toxicology 36, 104–116. 10.1016/j.reprotox.2012.11.011 23453003 PMC3587983

[B185] Tyebji S. Hannan A. J. Tonkin C. J. (2020). Pathogenic infection in male mice changes sperm small RNA profiles and transgenerationally alters offspring behavior. Cell Reports 31 (4), 107573. 10.1016/j.celrep.2020.107573 32348768

[B186] Vanselow J. Yang W. Herrmann J. Zerbe H. Schuberth H.-J. Petzl W. (2006). DNA-remethylation around a STAT5-binding enhancer in the αS1-casein promoter is associated with abrupt shutdown of αS1-casein synthesis during acute mastitis. J. Molecular Endocrinology 37 (3), 463–477. 10.1677/jme.1.02131 17170087

[B187] Walker S. Hartwich K. Seamark R. (1996). The production of unusually large offspring following embryo manipulation: concepts and challenges. Theriogenology 45 (1), 111–120. 10.1016/0093-691x(95)00360-k

[B188] WHO (2017). Don’t Pollute My Future! Impact Environment Children’s Health.

[B189] Wilkinson A. L. Zorzan I. Rugg-Gunn P. J. (2023). Epigenetic regulation of early human embryo development. Cell Stem Cell 30 (12), 1569–1584. 10.1016/j.stem.2023.09.010 37858333

[B190] Wu S. Li R. W. Li W. Li C.-j. (2012). Transcriptome characterization by RNA-seq unravels the mechanisms of butyrate-induced epigenomic regulation in bovine cells. PLoS One 7 (5), e36940. 10.1371/journal.pone.0036940 22615851 PMC3352864

[B191] Xiang T. Li L. Yin X. Zhong L. Peng W. Qiu Z. (2013). Epigenetic silencing of the WNT antagonist Dickkopf 3 disrupts normal Wnt/β‐catenin signalling and apoptosis regulation in breast cancer cells. J. Cellular Molecular Medicine 17 (10), 1236–1246. 10.1111/jcmm.12099 23890219 PMC4159020

[B192] Xu G. Chhangawala S. Cocco E. Razavi P. Cai Y. Otto J. E. (2020). ARID1A determines luminal identity and therapeutic response in estrogen-receptor-positive breast cancer. Nat. Genetics 52 (2), 198–207. 10.1038/s41588-019-0554-0 31932695 PMC7341683

[B193] Yan R. Cheng X. Gu C. Xu Y. Long X. Zhai J. (2023). Dynamics of DNA hydroxymethylation and methylation during mouse embryonic and germline development. Nat. Genetics 55 (1), 130–143. 10.1038/s41588-022-01258-x 36539615

[B194] Yang X.-J. Seto E. (2007). HATs and HDACs: from structure, function and regulation to novel strategies for therapy and prevention. Oncogene 26 (37), 5310. 10.1038/sj.onc.1210599 17694074

[B195] Yang X. Zhu H. Liu Y. Wang J. Song Y. Liao S. (2025). The biological function of genome organization. Int. J. Mol. Sci. 26 (18), 9058. 10.3390/ijms26189058 41009620 PMC12470977

[B196] Yao B. Christian K. M. He C. Jin P. Ming G.-l. Song H. (2016). Epigenetic mechanisms in neurogenesis. Nat. Reviews Neuroscience 17 (9), 537–549. 10.1038/nrn.2016.70 27334043 PMC5610421

[B197] Yehuda R. Daskalakis N. P. Bierer L. M. Bader H. N. Klengel T. Holsboer F. (2016). Holocaust exposure induced intergenerational effects on FKBP5 methylation. Biol. Psychiatry 80 (5), 372–380. 10.1016/j.biopsych.2015.08.005 26410355

[B198] Yeramilli V. Cheddadi R. Shah J. Brawner K. Martin C. (2023). A review of the impact of maternal prenatal stress on offspring microbiota and metabolites. Metabolites 13 (4), 535. 10.3390/metabo13040535 37110193 PMC10142778

[B199] Young L. E. Sinclair K. D. Wilmut I. (1998). Large offspring syndrome in cattle and sheep. Rev. Reproduction 3 (3), 155–163. 10.1530/ror.0.0030155 9829550

[B200] Zeineddine D. Abou Hammoud A. Mortada M. Boeuf H. (2014). The Oct4 protein: more than a magic stemness marker. Am. Journal Stem Cells 3 (2), 74–82. 25232507 PMC4163606

[B201] Zeng Y. Chen T. (2019). DNA methylation reprogramming during Mammalian development. Genes 10 (4), 257. 10.3390/genes10040257 30934924 PMC6523607

[B202] Zhang S. Rattanatray L. McMillen I. Suter C. Morrison J. (2011). Periconceptional nutrition and the early programming of a life of obesity or adversity. Prog. Biophysics Molecular Biology 106 (1), 307–314. 10.1016/j.pbiomolbio.2010.12.004 21168433

[B203] Zhang T. Cooper S. Brockdorff N. (2015). The interplay of histone modifications–writers that read. EMBO Reports 16 (11), 1467–1481. 10.15252/embr.201540945 26474904 PMC4641500

[B204] Zhang Y. Wang B. Sun W. Wang G. Liu Z. Zhang X. (2024). Paternal exposures to endocrine-disrupting chemicals induce intergenerational epigenetic influences on offspring: a review. Environ. International 187, 108689. 10.1016/j.envint.2024.108689 38688236

[B205] Zhou F. Wang R. Yuan P. Ren Y. Mao Y. Li R. (2019). Reconstituting the transcriptome and DNA methylome landscapes of human implantation. Nature 572 (7771), 660–664. 10.1038/s41586-019-1500-0 31435013

[B206] Zhu P. Guo H. Ren Y. Hou Y. Dong J. Li R. (2018). Single-cell DNA methylome sequencing of human preimplantation embryos. Nat. Genetics 50 (1), 12–19. 10.1038/s41588-017-0007-6 29255258

[B207] Zoubovsky S. P. Williams M. T. Hoseus S. Tumukuntala S. Riesenberg A. Schulkin J. (2022). Neurobehavioral abnormalities following prenatal psychosocial stress are differentially modulated by maternal environment. Transl. Psychiatry 12 (1), 22. 10.1038/s41398-022-01785-5 35039487 PMC8764031

[B208] Zucchi F. C. Yao Y. Ward I. D. Ilnytskyy Y. Olson D. M. Benzies K. (2013). Maternal stress induces epigenetic signatures of psychiatric and neurological diseases in the offspring. PLoS One 8 (2), e56967. 10.1371/journal.pone.0056967 23451123 PMC3579944

